# Revision of the species composition and distribution of Turkish sand flies using DNA barcodes

**DOI:** 10.1186/s13071-019-3669-3

**Published:** 2019-08-22

**Authors:** Ozge Erisoz Kasap, Yvonne-Marie Linton, Mehmet Karakus, Yusuf Ozbel, Bulent Alten

**Affiliations:** 1 Department of Biology, Ecology Section, Faculty of Science, VERG Laboratories, Hacettepe University, Ankara, Turkey; 2 Walter Reed Biosystematics Unit, Smithsonian Institution Museum Support Center, MRC-534, Suitland, MD 20746-2863 USA; 3 Department of Entomology, National Museum of Natural History, Smithsonian Institution, Washington, USA; 4 Department of Medical Microbiology, Faculty of Medicine, University of Health Sciences, Istanbul, Turkey; 5 Department of Parasitology, Faculty of Medicine, Ege University, Izmir, Turkey

**Keywords:** Phlebotomine, Sand fly, Turkey, *cox*1, Barcoding, Diversity

## Abstract

**Background:**

Currently, knowledge regarding the phlebotomine sand fly (Diptera: Psychodidae) fauna of Turkey is restricted to regions with endemic leishmaniasis. However, rapidly changing environmental and social conditions highlight concerns on the possible future expansion of sand fly-borne diseases in Turkey, promoting risk assessment through biosurveillance activities in non-endemic regions. Traditional morphological approaches are complicated by extensive cryptic speciation in sand flies, thus integrated studies utilizing DNA markers are becoming increasingly important for correct sand fly identification. This study contributes to the knowledge of the sand fly fauna in understudied regions of Turkey, and provides an extensive DNA barcode reference library of expertly identified Turkish sand fly species for the first time.

**Methods:**

Fly sampling was conducted at 101 locations from 29 provinces, covering all three biogeographical regions of Turkey. Specimens were morphologically identified using available keys. Cytochrome *c* oxidase I (*cox*1) barcode sequences were analyzed both for morphologically distinct species and those specimens with cryptic identity. A taxon identity tree was obtained using Neighbor Joining (NJ) analysis. Species boundaries among closely related taxa evaluated using ABGD, Maximum Likelihood (ML) and haplotype network analyses. Sand fly richness of all three biogeographical regions were compared using nonparametric species richness estimators.

**Results:**

A total of 729 barcode sequences (including representatives of all previously reported subgenera) were obtained from a total of 9642 sand fly specimens collected in Turkey. Specimens belonging to the same species or species complex clustered together in the NJ tree, regardless of their geographical origin. The species delimitation methods revealed the existence of 33 MOTUs, increasing the previously reported 28 recorded sand fly species by 17.8%. The richest sand fly diversity was determined in Anatolia, followed by the Mediterranean, and then the Black Sea regions of the country.

**Conclusions:**

A comprehensive *cox*1 reference library is provided for the sand fly species of Turkey, including the proposed novel taxa discovered herein. Our results have epidemiological significance exposing extensive distributions of proven and suspected sand fly vectors in Turkey, including those areas currently regarded as non-endemic for sand fly-borne disease.

**Electronic supplementary material:**

The online version of this article (10.1186/s13071-019-3669-3) contains supplementary material, which is available to authorized users.

## Background

Phlebotomine sand flies (Diptera: Psychodidae) are the only proven vectors of leishmaniasis, a group of neglected tropical diseases affecting more than one million people globally each year. Depending on the causative *Leishmania* species and host’s immunity, the disease manifests itself mainly in two clinical forms: the self-healing cutaneous leishmaniasis (CL) and the potentially lethal visceral leishmaniasis (VL) that are endemic in 85 and 74 countries, respectively [1].

The large landmass of Turkey (783,562 km^2^), nestled between the temperate and subtropical regions of Europe and near Asia, comprises three distinct biogeographical regions, i.e. Anatolian, the Black Sea, and the Mediterranean, each providing suitable conditions both for sand fly survival and propagation of the etiological disease agents they transmit. Both CL and VL are regarded as endemic in Turkey, with 2000 and 27 average reported annual human infections, respectively [1]. Some 25,369 cases of CL were reported in Turkey between 2005 and 2017, with highest incidences recorded in the Anatolian (56.08%) and Mediterranean (43.86%) regions. Of the reported 207 VL cases in Turkey between 2005 and 2014, over 50% occurred in the Mediterranean region (Turkish Ministry of Health Records, unpublished). Biosurveillance studies undertaken by our team previously highlighted the presence of several phleboviruses, including sand fly fever Turkey virus and Toscana virus, circulating in both the Anatolian and Mediterranean regions [2]. Since 2000, intensive vector incrimination and bionomic studies have been conducted in certain leishmania transmission foci in the Anatolian and in the Mediterranean regions of Turkey [3–8]. Undiagnosed human cases are suspected in the Black Sea region [2] yet the sand fly fauna of this region, and many other localities in the Anatolian and Mediterranean regions, remains unstudied.

Morphological identification of sand flies is notoriously difficult, often requiring expert morphological assessment of slide-mounted and/or dissected specimens. The lack of diagnostic characters for females of particular subgenera (e.g. *Adlerius*) [9], phenotypic plasticity observed in different populations of some species [10, 11], and the commonality of closely-related, sympatric cryptic taxa further amplifies this problem, seriously impeding reliable vector incrimination efforts [12, 13]. These restrictions often result in pathogen screening of sand fly samples without a priori morphological examination of specimens, leading to unreliable vector incrimination [14].

DNA sequence data from several mitochondrial and nuclear loci have been introduced in several studies to mitigate the need for accurate, reliable sand fly identification methods for both taxonomic and epidemiological studies (see [15] for a review). Among these, the DNA barcoding region of the mitochondrial cytochrome *c* oxidase I (*cox*1) gene, previously proposed by Hebert et al. [16], has proven highly useful in the differentiation of sand fly species [17–20]. The increasing availability of DNA barcode reference sequences has allowed retrospective identification of infected sand fly specimens, permitting accurate vector incrimination in high-throughput sand fly biosurveillance studies for the first time [21, 22].

The sand fly fauna of Turkey totals some 28 species to date (Table 1), very few of which have associated DNA sequences. In order to generate a comprehensive *cox*1 DNA barcoding reference library for Turkish sand flies to facilitate future taxonomic and biosurveillance studies, DNA barcodes were generated from morphologically verified voucher specimens collected from a wide range of localities in all three biogeographical regions in the country. Furthermore, the DNA barcode sequences were used to estimate the comparative sand fly species richness of the three biogeographic regions, evaluate the gene flow between populations of widely distributed species and to determine potential cryptic speciation within the Turkish sand fly fauna. The results are discussed in the context of sand fly-borne disease transmission in the region.

**Table 1:** The sand fly species previously reported from Turkey and the number of specimens barcoded from the three biogeographical regions in this study

Taxon		Biogeographical region		
Subgenus *Phlebotomus* Rondani and Berte		Anatolian	Black Sea	Mediterranean
* Phlebotomus papatasi* (Scopoli, 1786)	✓	36	0	20
Subgenus *Paraphlebotomus* Theodor				
* Phlebotomus alexandri* Sinton, 1928	✓	5	1	24
* Phlebotomus caucasicus* Marzinowsky, 1917	✓	2	0	0
* Phlebotomus jacusieli* Theodor, 1947	✓	3	3	1
* Phlebotomus sergenti* Parrot, 1917	✓	15	1	24
* Phlebotomus similis* Perfiliev, 1963	×			
	*Paraphlebotomus* sp.	0	3	0
Subgenus *Larroussius* Nitzulescu				
* Phlebotomus burneyi* Lewis, 1967	*Phlebotomus kandelakii* (*s.l.*)	17	25	10
* Phlebotomus kandelakii* Schurenkova, 1929				
* Phlebotomus major* Annandale, 1910	*Phlebotomus major* (*s.l.*)	39	46	84
* Phlebotomus neglectus* Tonnoir, 1921				
* Phlebotomus syriacus* Adler & Theodor, 1931				
* Phlebotomus galilaeus* Theodor, 1958	*Phlebotomus perfilieiwi* (*s.l.*)	47	17	32
* Phlebotomus perfiliewi* Parrot, 1939				
* Phlebotomus transcaucasicus* Perfiliev, 1937				
* Phlebotomus tobbi* Adler, Theodor & Lourie, 1930	✓	39	15	53
Subgenus *Adlerius* Nitzulescu				
* Phlebotomus balcanicus* Theodor, 1958	✓	0	0	3
* Phlebotomus brevis* Theodor & Mesghali, 1964	✓	1	0	0
* Phlebotomus halepensis* Theodor, 1958	✓	17	8	0
* Phlebotomus kyreniae* Theodor, 1958	✓	5	0	0
* Phlebotomus simici* Nitzulescu, 1931	✓	8	0	9
	*Adlerius* spp.	33	4	13
Subgenus *Transphlebotomus* Adler				
* Phlebotomus anatolicus* Erisoz Kasap, Depaquit & Alten, 2015	✓	0	0	1
* Phlebotomus economidesi* Léger, Depaquit & Ferté, 2001	✓	0	0	0
* Phlebotomus killicki* Dvorak, Votypka & Volf, 2015	✓	0	0	1
* Phlebotomus mascittii* Grassi, 1908	×	0	0	0
Subgenus *Sergentomyia* França & Parrot				
* Sergentomyia antennata* Newstead, 1912	×	0	0	0
* Sergentomyia dentata* Sinton, 1933	✓	0	0	28
* Sergentomyia minuta* (Rondani, 1843)	✓	1	0	34
* Sergentomyia theodori* (Parrot, 1942)	×	0	0	0

*Key*: ✓ indicates the sand fly species previously reported from Turkey and were sampled in this study;× indicates the sand fly species previously reported from Turkey but were not sampled during this study

## Methods

### Sampling, specimen identification and sequencing

**Fig. 1:**
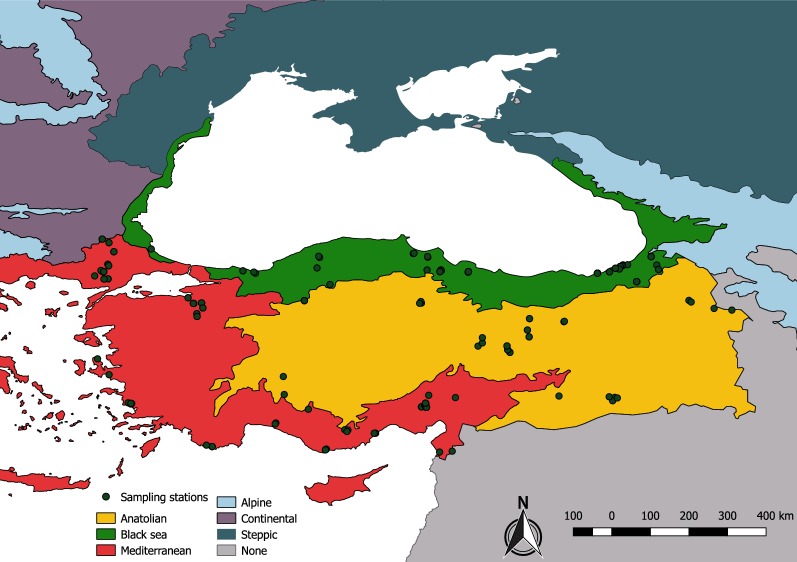
Map indicating the sand fly collection sites and the biogeographical regions of Turkey and the neighboring European countries

Between 2005 and 2016, sand flies were collected at 101 locations from 29 provinces in all three biogeographical regions of Turkey (Anatolia, Mediterranean and Black Sea regions). The European Environment Agency guidelines [23] were followed for delimiting the borders of these regions, and the geographical coordinates of the collection localities were visualized in QGIS 2.18.0 (Fig. 1). Adult sand flies were mainly collected using CDC miniature light traps placed in or around domestic animal enclosures closely associated with human dwellings. Depending on the diversity of probable host species and the accessibility of the animal enclosures, one to six traps were set in each location (Additional file 1: Table S1). As the climatic conditions within the same biogeographical region vary widely, the complete developmental threshold degree-day value obtained for *Phlebotomus papatasi* [24] was utilized to estimate the optimal period of the season for adult emergence for each province. Previously published field data [4, 7, 49] were also considered to optimize the success of the field collections.

Following collection, specimens were stored in 96% ethanol for further analyses. The head and the terminal 2–3 abdominal segments of each specimen were dissected and mounted for morphological identification using the keys available for the adult Old World sand flies [9, 25, 26] while the thorax and the rest of the abdomen was retained in ethanol for DNA extraction. Where possible, ten specimens representing both males and females of each species were selected for DNA barcoding from each province. Additionally, specimens that could only be morphologically assigned to subgenera were also included in the analysis.

DNA was obtained from individual sand flies following a previously documented extraction protocol [12]. The universal LCO1490 and HCO2198 primers [27] were used to amplify the ~650 bp barcoding region of the *cox*1 gene, using previously defined PCR reaction conditions optimized in our laboratory [28]. Bi-directional sequencing of the purified amplification products was performed using the original PCR primers at RefGen Gene Research and Biotechnology Company (Ankara, Turkey).

### Data analysis

The resultant *cox*1 sequences were aligned using the ClustalW Multiple Alignment algorithm, as implemented in the BioEdit v.7.2.5 [29] alignment editor. Neighbor-Joining (NJ) analysis was conducted on 1000 replicates under the assumptions of Kimura’s two parameter (K2P) substitution model in MEGA v.6.0 [30] in order to obtain a robust taxon identity tree with bootstrapped branch support.

The K2P distances generated through pairwise sequence comparisons using the default settings of *P-*values (0.1–0.001) in the Automatic Barcode Gap Discovery (ABGD) web-interface program (http://wwwabi.snv.jussieu.fr/public/abgd/) [31] were used to assign each barcode sequence into molecular operational taxonomic units (MOTUs). ABGD splits the sequences into groups through two subsequent partitioning. Using a user-defined range of prior intraspecific distance divergence threshold values (*P*), this method first infers a barcode gap and uses this gap value to partition the data into clusters. In the second step, these clusters are repeatedly partitioned into hypothetical species until no more splits take place [32].

Species boundaries among closely related taxa within each subgenus were further evaluated by Maximum Likelihood (ML) analysis in MEGA v.6.0, using only the unique representative haplotypes, as identified by DnaSP v.5.10.01 [33]. The best-fit evolutionary model for each sub data set was defined using the Smart Model Selection (SMS) software [34]. Parsimony networks using TCS method [35] generated in PopArt [36] and compared with the results obtained by ABGD.

### Species richness estimates within biogeographical regions

As the number of traps set and the number of individuals selected for DNA barcoding differed in each biogeographical region, we built provisional species accumulation curves to evaluate the completeness of our sampling effort in EstimateS v.9.1.0. [37]. Using the frequency data of rare species in an assemblage, non-parametric species richness estimators allow estimates of the total species richness, even when the sampling efforts are not complete. The mean values of incidence-based richness estimator Chao2 and the 95% confidence intervals in EstimateS v.9.1.0. were used for the comparison of biogeographical regions in terms of MOTU richness.

## Results

A total of 9642 sand flies were collected during the field activities and sand flies were collected at all of the 101 sampling locations, spanning 29 provinces. The morphological analyses of the specimens showed that 15 species and four species complexes (around 90% of the previously documented sand fly fauna of Turkey) were sampled during the study period. *cox*1 barcode sequences were obtained from 729 specimens, expertly identified as representatives of each traditionally recognized taxon. Specimens belonging to known species complexes (as *sensu lato*, hereinafter *s.l.*), as well as the 50 morphologically indistinguishable females of the subgenus *Adlerius* and three unidentified *Paraphlebotomus* female specimens, were included in the analyses. The sand fly species previously reported from Turkey and the number of specimens barcoded from each of the three biogeographical regions are shown in Table 1.

After trimming and alignment, the length of the target *cox*1 sequences ranged from 547 to 630 base pairs. As there were no gaps and stop codons, the data set was revealed to be free of pseudogenes and nuclear insertions of mitochondrial DNA. The NJ analysis of the data revealed that all specimens morphologically determined as belonging to the same species or species complex clustered together, irrespective of geographical origin. Barcode sequences obtained for the 50 unidentifiable *Adlerius* spp. females allowed retrospective species confirmation of most of these specimens, as they grouped together with their conspecific males. However, a group of females (*n* = 13) were found to have deeply diverged from all other species analyzed within the subgenus *Adlerius*, suggesting the presence of another species. Similarly, the three morphologically unidentified *Paraphlebotomus* females did not adhere to any morphologically verified clusters (Additional file 2: Figure S1).

Using the prior intraspecific divergence threshold value of 1.29%, ABGD assigned the 742 *cox*1 barcode sequences into 34 distinct groups. Specimens conforming to the morphologically verified species, *Phlebotomus papatasi*, *P. caucasicus*, *P. jacusieli*, *P. perfiliewi* (*s.l.*), *P. tobbi*, *P. balcanicus*, *P. brevis*, *P. halepensis*, *P. kyreniae*, *P. simici*, *P. anatolicus*, *P. economidesi* and *P. killicki*, were grouped in individual MOTUs. However, deep intraspecific divergences (mean K2P distance > 0.25) within *P. alexandri*, *P. sergenti* (*s.l.*), *P. kandelakii* (*s.l.*), *P. major* (*s.l.*), *Sergentomyia dentata* and *S. minuta* resulted in the representation of at least two MOTUs in each of these taxa. Two distinct groups were detected within *P. alexandri* and *S. dentata*, while *P. kandelakii* (*s.l.*), *P. sergenti* (*s.l.*) and *S. minuta* were split into three barcode clusters each. With a mean intraspecific K2P distance of 2.7%, *P. major* (*s.l.*) was shown to comprise six distinct groups. In concordance with the results obtained from NJ analysis, the unidentified females of *Adlerius* sp. (*n* = 13) and *Paraphlebotomus* sp. (*n* = 3) were each grouped in two unique MOTUs.

### Subgenus *Phlebotomus*

**Fig. 2:**
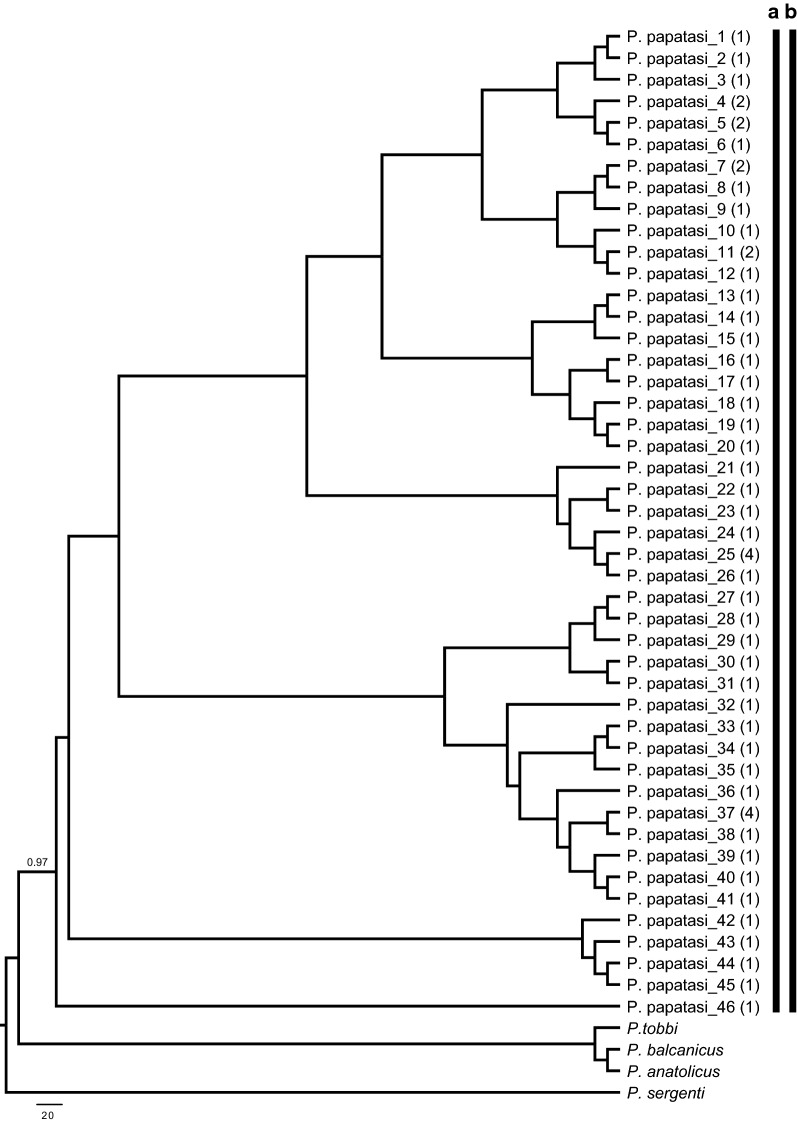
Maximum Likelihood (ML) tree constructed for the 46 *P. papatasi* haplotypes (GenBank accession numbers: MN086366–MN086411) based on mitochondrial *cox*1 gene region. Bars representing the MOTUs inferred from ABGD (**a**) and TCS algorithms (**b**). The bootstrap values higher than 50% are indicated

DNA barcode sequences were recovered for 56 specimens of *P. papatasi*, the only species of subgenus *Phlebotomus* recorded from Turkey. *Phlebotomus papatasi* is common in the Anatolian and Mediterranean regions but was not detected in collections from the Black Sea region. The final alignment was 630 bp long, and the total number of haplotypes was 46 with a haplotype diversity (Hd) of 0.989 (GenBank: MN086366–MN086411). The ML analysis of the *P. papatasi* haplotypes under the T92+G model revealed the same results obtained by ABGD, with all sequences grouped in a single clade (Fig. 2). Similarly, TCS identified a single network for all the *P. papatasi* sequences analyzed. As there were no *P. papatasi* specimens collected from the Black Sea region, all the haplotypes were generated from representative specimens collected in the Anatolian and Mediterranean regions. Three haplotypes (*P. papatasi*-11, P. *papatasi*-25 and *P. papatasi*-37) were common to both biogeographical regions (Additional file 3: Figure S2).

### Subgenus *Paraphlebotomus*

**Fig. 3:**
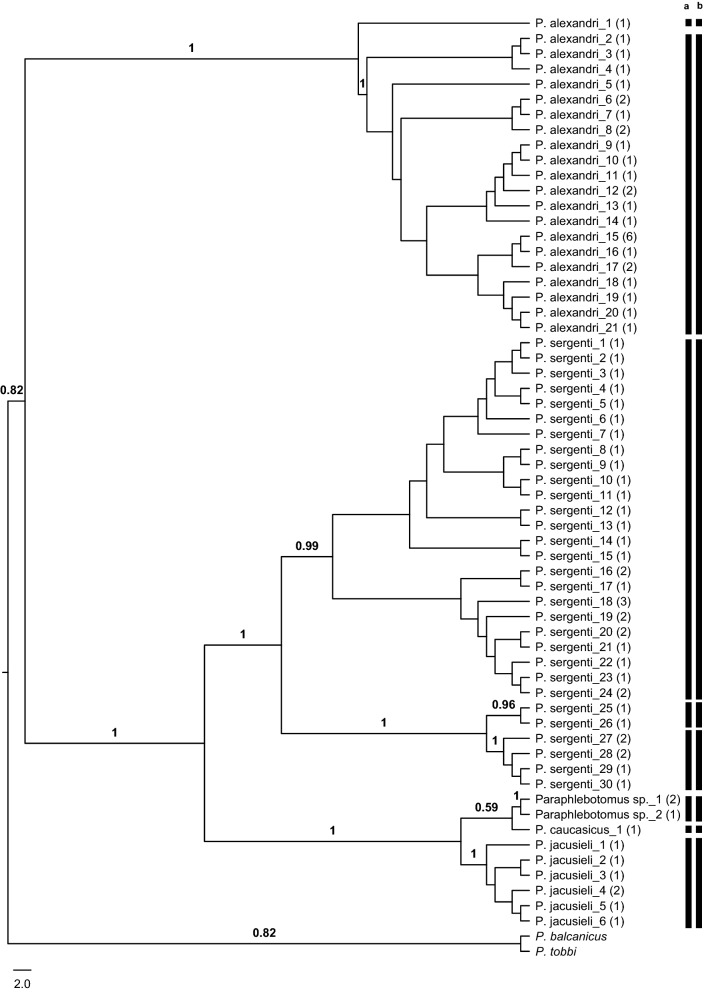
Maximum Likelihood (ML) tree constructed for the 60 haplotypes belonging to subgenus *Paraphlebotomus* (GenBank: MN086412–MN086471) based on mitochondrial *cox*1 gene region. Bars represent the MOTUs inferred from ABGD (**a**) and TCS (**b**) algorithms. The bootstrap values higher than 50% are indicated

The aligned matrix of 83 *cox*1 barcodes obtained for species of subgenus *Paraphlebotomus* consisted of 603 characters. The 83 specimens were represented by 60 unique *cox*1 haplotypes (GenBank: MN086412–MN086471) (Hd = 0.989) that fell in two major clades after the ML analysis conducted under the GTR+G+I substitution model. Clade 1 comprised of *P. alexandri* haplotypes grouped in two well supported lineages. The first lineage was represented with the *P. alexandri*-1 haplotype, while the rest of the 20 haplotypes was grouped in the second lineage (Fig. 3). In concordance with the ABGD, parsimony network analysis identified two independent networks for *P. alexandri* data set that also match with the two lineages recovered in the ML analysis. The first network comprised the haplotypes sampled from the three biogeographical regions and three of them (*P. alexandri*-6, *P. alexandri*-15 and *P. alexandri*-17) were shared among the Anatolian and the Mediterranean regions. Originating from the Anatolian region, *P. alexandri*-1 was the only representative of the second network, although one other haplotype (*P. alexandri*-13) from the same location was placed in the first network (Additional file 4: Figure S3). The mean K2P distance between these two lineages (7.5%) was comparable with those of morphologically distinguishable species pairs, e.g. *P. brevis* × *P. simici* (5.5%) and *P. caucasicus *× *P. jacusieli* (4.3%) (Additional file 5: Table S2), suggesting this taxon comprises two distinct species in Turkey.

The second clade within the subgenus *Paraphlebotomus* was comprised of the well supported lineages representing *P. sergenti* (*s.l.*), *P. caucasicus*, *Paraphlebotomus* sp. and *P. jacusieli* (Fig. 3). Supporting the recursive partitioning of the data by ABGD, *P. sergenti* (*s.l.*) was divided into three lineages, also defined as three independent networks by TCS. Most of the haplotypes originating from the Mediterranean region as well as all the haplotypes from the Black Sea and the Anatolian region were grouped in the first network. Only one haplotype, *P. sergenti*-18, within this network was shared between the Anatolian and the Mediterranean regions. Six haplotypes from the same province of the Mediterranean region were grouped in the second and third networks that are concordant with the second and third lineages in the ML tree (Additional file 4: Figure S3). The mean K2P distances between the three lineages of *P. sergenti* (*s.l.*) were found to be substantially higher (2.7–5.0%) than those yielded for within these lineages (0.2–1.1%) (Additional file 5: Table S2). The two specimens of Anatolian *P. caucasicus* were represented by a single haplotype that was clearly diverged from the morphologically unidentified *Paraphlebotomus* sp. haplotypes from the Black Sea region (mean interspecific K2P distance = 3.7%). Despite its low numbers in catches (*n* = 7), *P. jacusieli* appears to be widely distributed, and was placed as the sister species of *P. caucasicus* and *Paraphlebotomus* sp. in the ML tree. Similarly, TCS sorted these three taxa in distinct networks (Additional file 4: Figure S3).

### Subgenus *Larroussius*

**Fig. 4:**
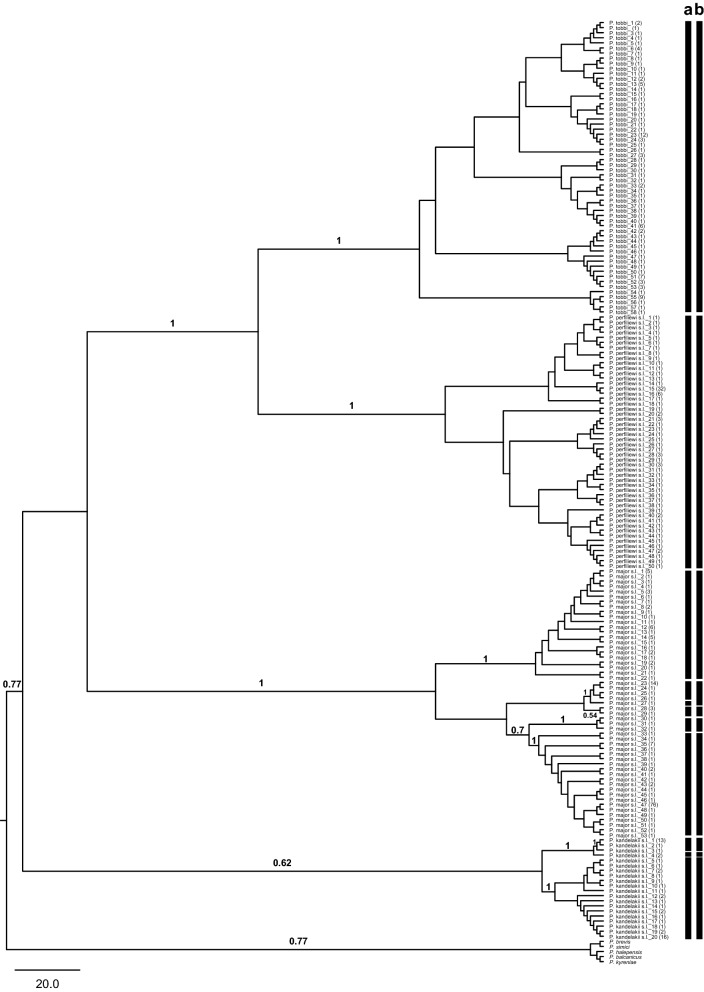
Maximum Likelihood (ML) tree constructed for the 181 haplotypes belonging to subgenus *Larroussius* (GenBank: MN086472–MN086652) based on mitochondrial *cox*1 gene region. Bars represent the MOTUs inferred from ABGD (**a**) and TCS (**b**) algorithms. The bootstrap values higher than 50% are indicated

In total, 424 *cox*1 barcode sequences were obtained for the *Larroussius* species distributed in Turkey. Some 181 unique haplotypes (Hd = 0.956) were detected in the 594 base pair aligned sub-set (GenBank: MN086472–MN086652). The ML analysis conducted under the GTR substitution model separated all the morphologically recognized taxa clearly (Fig. 4). In concordance with the ABGD results, *P. tobbi* haplotypes were grouped together in a single clade. This was further supported by TCS that linked all the geographically distinct haplotypes of *P. tobbi* in a single network. Three haplotypes were shared across all three biogeographical regions (*P. tobbi*-41, *P. tobbi*-51 and *P. tobbi*-53), while three others (*P. tobbi*-27, *P. tobbi*-52 and *P. tobbi*-55) were present only in the Anatolian and the Mediterranean regions. Both *P. tobbi*-1 and *P. tobbi*-13 haplotypes were found only in the Anatolian and the Black Sea regions (Additional file 6: Figure S4).

The 96 *P. perfiliewi* (*s.l.*) barcodes (50 haplotypes) formed a single group in the ML tree, congruent with both the recursive partitioning of the complete data set by ABGD, as well as the single network identified by the parsimony network analysis. *P. perfiliewi* (*s.l.*)-15 and *P. perfiliewi* (*s.l.*)-16 were the most common haplotypes in the network, (Additional file 7: Figure S5).

The grouping pattern of the 53 haplotypes produced for 169 *P. major* (*s.l.*) specimens in the ML tree was reflective of the results obtained by the TCS. The first network comprised the haplotypes from all the three biogeographical regions with *P. major*-5 shared between all the regions and *P. major*-1 occurring both in the Anatolian and in the Mediterranean regions. The second network included the haplotypes only from the Mediterranean region, with *P. major*-23 identified as the most frequent haplotype in this network. With a restricted distribution in the Anatolian region, *P. major*-28 and *P. major*-29 were grouped in the third network, while two haplotypes from the Black Sea region (*P. major*-30 and *P. major*-32) and one from the Anatolian region (*P. major*-31) were included in the fourth network. The final network was consisted of the haplotypes distributed in all the three biogeographical regions. The haplotype *P. major*-47 was the most common, present in all regions, but predominated in the Mediterranean. Two haplotypes (*P. major*-35 and *P. major*-43) are shared in the Anatolian and the Mediterranean regions (Additional file 8: Figure S6). The mean K2P distances between the five lineages recovered in the ML analysis ranged between 4.1–9.1% (Additional file 5: Table S2). The barcode clusters generated by ABGD were in concordance with the ML and TCS results, except one haplotype (*P. major*-27) grouped in the second network was assigned to an independent cluster.

Three lineages were identified within the 20 *P. kandelakii* (*s.l.*) haplotypes included in the ML analysis. The mean K2P distances between these lineages (2.4–8.6%) were higher than those yielded for within lineages (0.00–0.07%). Parsimony network analysis generated three independent networks concordant with the distinct lineages of *P. kandelakii* (*s.l.*) in the ML tree. The first two networks included the haplotypes originating from specimens from the Anatolian region, while the third comprised the haplotypes distributed both in the Black Sea and the Mediterranean regions. No haplotypes were shared between regions (Additional file 9: Figure S7).

### Subgenus *Adlerius*

**Fig. 5:**
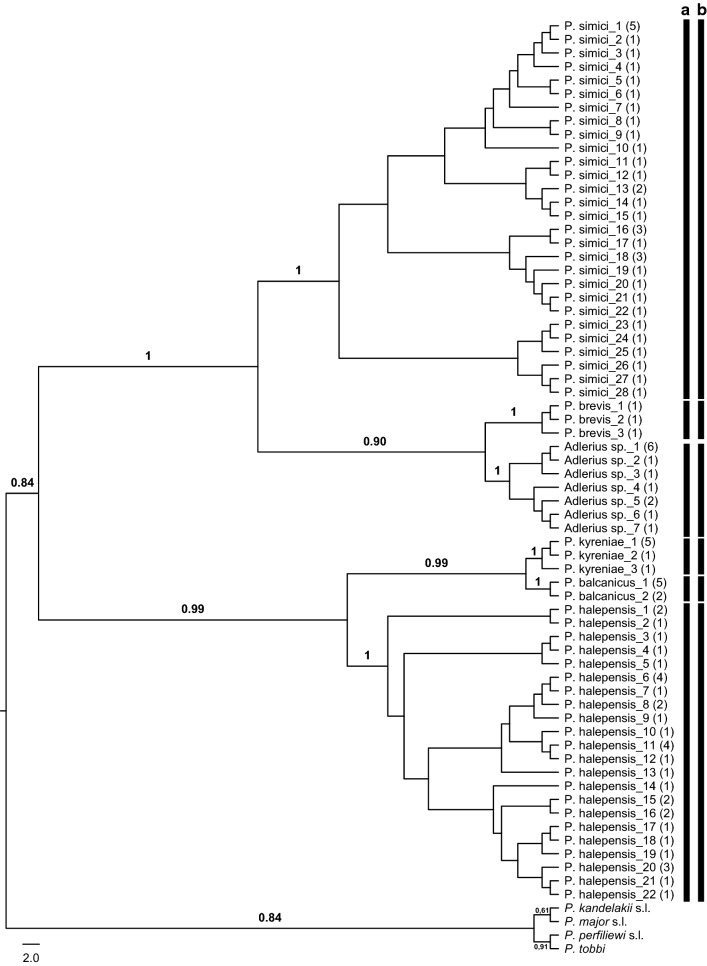
Maximum Likelihood (ML) tree constructed for the 65 haplotypes belonging to subgenus *Adlerius* (GenBank: MN086653–MN086717) based on mitochondrial *cox*1 gene region. Bars represent the MOTUs inferred from ABGD (**a**) and TCS (**b**) algorithms. The bootstrap values higher than 50% are indicated

*cox*1 barcodes were obtained for all *Adlerius* species reported from Turkey to date. The length of the final alignment was 547 bp, and a total of 65 haplotypes were produced with a haplotype diversity (Hd) of 0.985 (GenBank: MN086653–MN086717). Congruent with the ABGD results, the ML analysis conducted under the GTR+G+I model recovered six well supported lineages located in two main clades (Fig. 5). Distributed in the Anatolian and Black Sea regions, *P. simici* represented the first lineage of the *Adlerius* phylogeny. A single network was identified for the 37 haplotypes; haplotypes *P. simici*-1 and *P. simici*-16 were shared between the two regions (Additional file 10: Figure S8). Three haplotypes of *P. brevis* and seven haplotypes of unidentified *Adlerius* sp. were grouped in two distinct lineages and placed as the sister taxa of *P. simici* in the first clade. Similarly, the parsimony network analysis grouped the *P. brevis* and *Adlerius* sp. haplotypes in two independents networks, the former being restricted in the Mediterranean region while the latter occurring only in the Anatolian region (Additional file 10: Figure S8).

The second clade included three lineages that correspond to *P. kyreniae*, *P. balcanicus*, and *P. halepensis. Phlebotomus kyreniae* was represented by three haplotypes from the Anatolian region, whereas *P. balcanicus* lineage included only two haplotypes from the Mediterranean region. Distributed both in the Anatolian and in the Black Sea regions, *P. halepensis* appeared as the sister species of *P. balcanicus* and *P. kyreniae* in the second clade of the ML tree. TCS identified unique networks for each of these species (Additional file 10: Figure S8).

### Subgenus *Transphlebotomus*

**Fig. 6:**
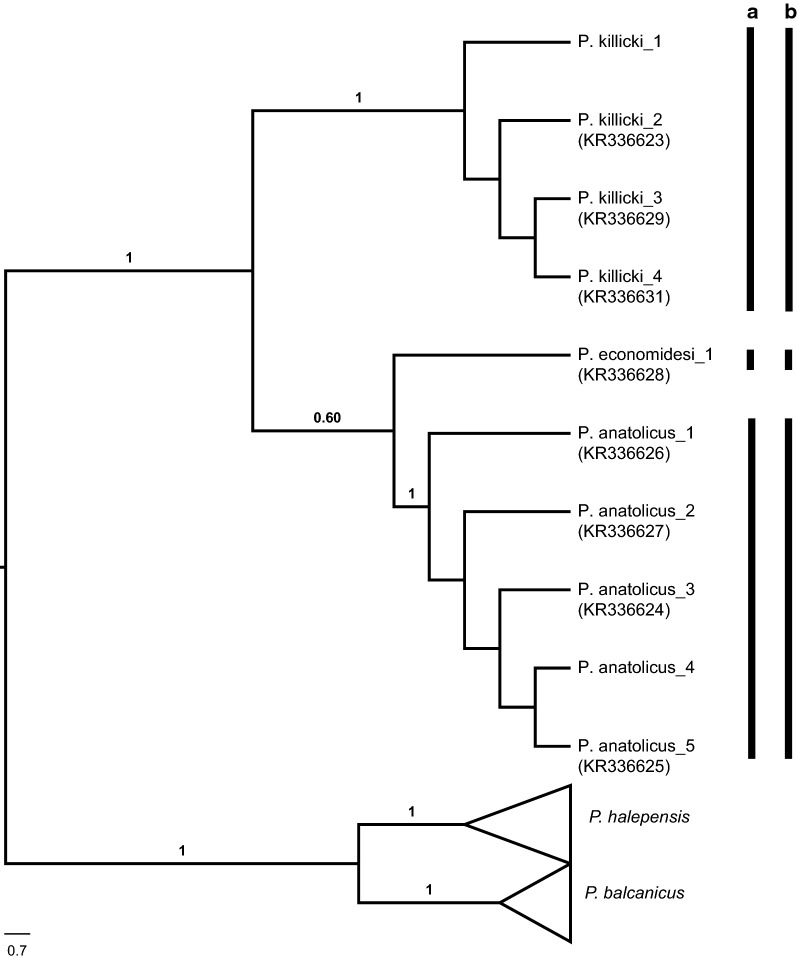
Maximum Likelihood (ML) tree constructed for the 10 haplotypes belonging to subgenus *Transphlebotomus* (GenBank: MN086718–MN086719) based on mitochondrial *cox*1 gene region. Previously published sequences appear with corresponding GenBank accession numbers. Bars represent the MOTUs inferred from ABGD (**a**) and TCS (**b**) algorithms. The bootstrap values higher than 50% are indicated

The internal systematics of the subgenus *Transphlebotomus* using multi-locus DNA regions (including *cox*1) have been previously assessed [18]. Here, two additional *cox*1 sequences (GenBank: MN086718–MN086719) generated from females collected in the Mediterranean region were compared with our previously published sequences (GenBank: KR336623–KR336631), and were identified as *P. anatolicus* and *P. killicki.* ABGD assigned the *Transphlebotomus* haplotypes into three groups, in concordant with the ML (using the GTR+G substitution model) and parsimony network analyses, and with our previous findings (Fig. 6, Additional file 11: Figure S9).

### Subgenus *Sergentomyia*

**Fig. 7:**
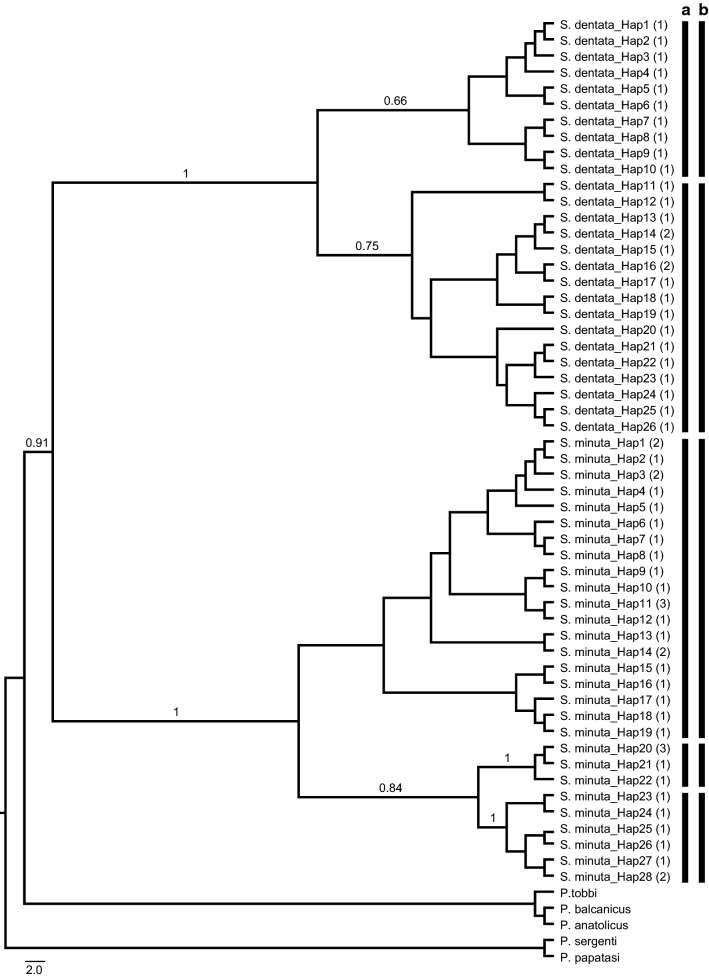
Maximum Likelihood (ML) tree constructed for the 54 haplotypes belonging to *Sergentomyia* subgenus (GenBank: MN086720–MN086773) based on mitochondrial *cox*1 gene region. Bars represent the MOTUs inferred from ABGD (**a**) and TCS (**b**) algorithms. The bootstrap values higher than 50% are indicated

In total, 63 *cox*1 sequences were generated from two morpho-species of *Sergentomyia* from Turkey, resulting in 54 haplotypes (Hd = 0.99) in the final 607 bp alignment (GenBank: MN086720–MN086773). The ML analysis conducted using the T93+G substitution model resulted in two well-supported clades. The first clade included Mediterranean *S. dentata* populations which divided into two distinct lineages; these groupings were supported by the two independent networks recovered in the TCS analysis and two unique barcode clusters generated by the ABGD. The second clade comprised the *S. minuta* haplotypes grouped into three lineages with varying bootstrap supports, congruent with the recursive partitioning results of ABGD and independent networks generated by TCS. With the exception of *S. minuta*-2 from the Anatolian region, all haplotypes were restricted in the Mediterranean region (Fig. 7, Additional file 12: Figure S10).

### Species richness estimates

We assessed the completeness of our sampling effort using the accumulation curve for all 718 barcode sequences and 33 MOTUs recovered using all three methodologies (ABGD, ML and parsimony network analyses). The barcode sequences of 24 *P. major* (*s.l.*) specimens were excluded in this analysis as we only used these specimens for molecular identification and do not have any data regarding the species composition of the localities from where they were collected.

**Fig. 8:**
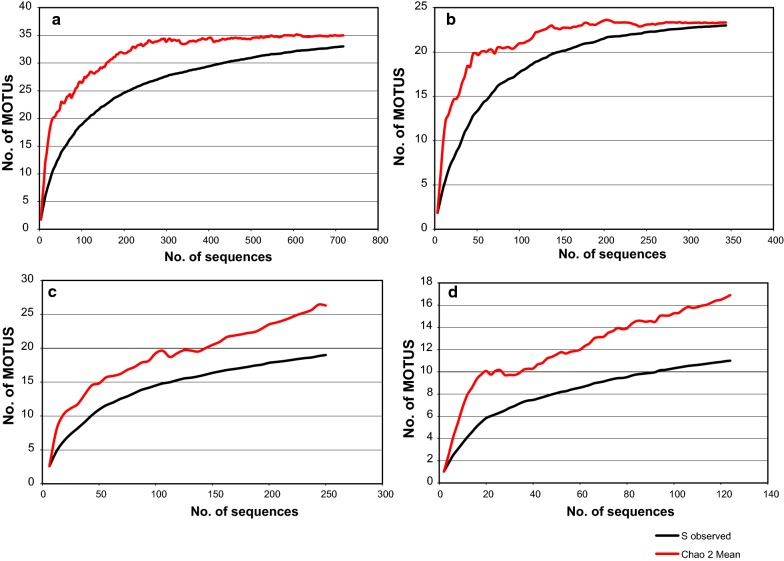
MOTU accumulation curves for **a** Turkey (complete data set), **b** the Mediterranean biogeographical region, **c** the Anatolian biogeographical region and **d** the Black Sea biogeographical region

The Chao2 estimator proposed 34.99 (95% CI: 33.28–46.91) sand fly species in Turkey and the incidence-based accumulation curve for the whole dataset suggested that our sampling was almost complete. Although the accumulation curve for sand flies of the Mediterranean region reached an asymptote, indicating the fauna is well sampled, the species richness of the Anatolian and the Black Sea regions requires more sampling (Fig. 8). For the Mediterranean region, the Chao2 estimator of 23.33 (95% CI: 23.02–28.92) closely aligned with the 23 MOTUs revealed in our analysis. However, the Chao2 estimates of 26.31 (95% CI: 20.29–60.54) and 16.9 (95% CI: 11.93–48.35) for the Anatolian and the Black Sea regions, respectively, were much higher than the 19 and 11 MOTUs we determined in our field collections, suggesting our sampling here is far from complete. The distribution of the MOTUs observed in the three biogeographical regions is illustrated in Additional file 13: Figure S11.

## Discussion

Accurate identification of sand fly specimens is critical to better understanding of sand fly-pathogen interactions, correct determination of associated bionomics and substantiated risk assessments of sand fly-transmitted diseases. Morphological identification remains the keystone of sand fly taxonomy, but the integration of molecular data is vital in solving taxonomic problems and determining species-diagnostic bionomic traits. To our knowledge, this study comprises the first comprehensive attempt to determine the utility of DNA barcoding for the robust identification of Turkish sand fly fauna, and expands our knowledge of sand fly composition and distribution in previously unstudied regions of the country. Furthermore, this study provides an extensive morphologically-verified DNA barcode reference library, which will be of interest to sand fly researchers in Europe and in the Middle East.

Prior to this study, 28 species were reported in Turkey, representing two genera and six subgenera. *Phlebotomus papatasi*, the vector of *L. major* in North Africa and the Middle East, is the sole representative of the subgenus *Phlebotomus* in Turkey. Irrespective of species delimitation algorithm applied, *P. papatasi* specimens collected from the Anatolian and the Mediterranean regions appeared in one cluster, supporting earlier studies that showed continuous gene flow between the distinct geographical populations over a wide distributional range [38, 39].

Most Turkish *Paraphlebotomus* sand flies are proven vectors of leishmaniasis in the Old World. *Phlebotomus alexandri* (distributed from Spain across to China and down to southern Ethiopia) is responsible for transmitting pathogens in the *Leishmania donovani* complex [40]. Two highly divergent lineages were detected in Turkish *P. alexandri* samples: the first exhibited shared haplotypes between all three regions, and low mean intra-lineage genetic distances (0.4%), indicative of continual interchange between populations. The second lineage represented by a single Anatolian sample collected in sympatry with *P. alexandri* lineage 1, showed 7.5% sequence divergence from the first, highly suggestive of cryptic speciation within this taxon in Turkey. Comparison of all publically available *cox*1 barcodes of *P. alexandri* from different countries determined that the most common Turkish haplotype clustered with one specimen from Israel (GenBank: KF483668), while the unique, highly divergent specimen grouped readily with *P. alexandri* specimens from Algeria (GenBank: KJ481082, KJ481085, KJ481092–KJ481093, KJ481095) and China (KF137558–KF137559). Sequences from topotypic *P. alexandri* are essential to ascertain the true identity of *P. alexandri* (*s.s.*) and more specimens are needed to evaluate the diversification of this taxon across its range.

*Phlebotomus* (*Paraphlebotomus*) *sergenti* (*s.l.*) is the proven vector of *Leishmania tropica* in several Middle Eastern and African countries, as well as in Turkey [3, 40]. Significant morphological variation has been reported in *P. sergenti* across its Old World distribution, and it has long been suggested that *P. sergenti* comprises a cryptic species complex [41]. Several genetic studies have reported distinct mitochondrial and nuclear lineages that do not correlate with the proposed variant morphotypes [10, 41–43]. In Turkey, the widely distributed *P. sergenti* (*s.l.*) comprises three distinct lineages (2.7–5.0% *cox*1 sequence differences), the first of which is ubiquitous across Turkey and clusters with Algerian samples (GenBank: KJ481066–KJ481068, KJ481071) [43], and the second and third lineages restricted to one province located in the northwestern part of the Mediterranean region. Although a closely related taxon, *Phlebotomus similis*, has been also reported in Turkey, its presence is controversial. None of the *P. sergenti* lineages sequenced here matched *P. similis* sequences available from Crete, suggesting these two additional lineages may represent additional biodiversity within the previously defined *P. sergenti* species complex [44].

*Phlebotomus* (*Paraphlebotomus*) *caucasicus*, the vector of *L. major* in Afghanistan and the suspected vector of zoonotic cutaneous leishmaniasis in Iran, has been recently reported from the Northeastern Anatolian region. We collected this species only in close proximity to the original report, and in low numbers, suggesting this is a rare species. *cox*1 barcodes (*n* = 2) generated here clearly differentiated this taxa from other *Paraphlebotomus* species with mean genetic distances of 4.3–18.3%. A group of unconfirmed *Paraphlebotomus* sp. females in the Black Sea region were placed as the sister species of *P. caucasicus* in the ML analysis and regarded unique by ABGD and TCS algorithms. Re-examination of the voucher slides confirmed unique characters of the spermatheca, further evidencing this hitherto undescribed diversity within *P. caucasicus*.

The non-vector *Phlebotomus* (*Paraphlebotomus*) *jacusieli* is widely distributed in the Middle East and Caucasia, and has previously been reported in the Anatolian and Mediterranean regions of Turkey. Here, to the best of our knowledge, we document its presence in the Black Sea Region for the first time.

Species of the subgenus *Larroussius* are regarded as the main vectors of *L. infantum* in the Mediterranean. *Phlebotomus tobbi* is the proven vector of *L. infantum* in the Mediterranean region of Turkey [5, 45] and in Cyprus [46], and is the suspected vector in Albania, Greece and Iran [40, 47, 48]. Our studies show that *P. tobbi* is a single abundant, genetically diverse (58 *cox*1 haplotypes in 107 samples sequenced) species, which is common across all three biogeographical regions of Turkey. Analysis of publicly available *P. tobbi* samples from Cyprus (GenBank: KX826025, KX826041), Greece (GenBank: KU519502, KU519503, KT634317) and Israel (GenBank: KF483675) further confirms that *P. tobbi* comprises a single, widely-dispersed species across the Mediterranean Basin, without evidence of significant geographical structuring.

Populations of *P.* (*Larroussius*) *perfiliewi* (*s.l.*) are proven or suspected vectors of *L. infantum* in Europe and in the Middle East. Three essentially allopatric species (*P. galilaeus*, *P. perfiliewi* and *P. transcaucasicus*) comprise the *P. perfiliewi* complex; females are isomorphic, and male characteristics appear to vary by geographical origin [11]. *Phlebotomus perfiliewi* is the most widely distributed species with records from Crimea to North Africa and in many European countries, *P. galilaeus* is reported from Cyprus and Israel, and *P. transcaucasicus* is restricted to Caucasia and Central Asia. Turkey is the only known country where these species purportedly occur in sympatry [4, 6, 49]. However, in the 96 *P. perfiliewi* specimens sampled herein, all were identified as a single taxon (*P. perfiliewi*) in all three species delimitation methods, with a mean genetic distance of 1.1%.

The *Phlebotomus* (*Larroussius*) *major* complex comprises six morphologically similar and largely allopatric species (*P. neglectus*, *P. notus*, *P. major*, *P. syriacus*, *P. wenyoni* and *P. wui*). *Phlebotomus neglectus* and *P. syriacus* are only sympatric in the Middle East, including Turkey [12, 50]. Previously, our studies confirmed this sympatry and suggested the existence of a possible third taxon, using genetic data from the mitochondrial cytochrome oxidase b (Cyt-b) gene and the nuclear marker elongation factor (EF-1α) [12]. Here we confirm the extensive geographical distribution of *P. major* (*s.l.*) throughout the country, and further confirm the existence of the previously described members of this species complex (*P. neglectus*, *P. syriacus* and the third provisional taxon) and the distribution of two additional lineages of *P. major* complex in Turkey. *cox*1 barcode sequences of 169 *P. major* (*s.l.*) specimens from across Turkey were evaluated, including those specimens analyzed in the previous study [12]. Corresponding to the first lineage of the ‘Major Group’ in the ML tree of the subgenus *Larroussius* (Fig. 4), *P. neglectus* is widely distributed, occurring in all three biogeographical regions of Turkey. Supporting our previous results, the distribution of *P. syriacus* (lineage 2 herein) is restricted within the southeastern parts of the Mediterranean region while the fifth lineage of the *P. major* complex (corresponding to the third proposed taxon in our previous study) has an extensive distribution throughout the country. The newly described third lineage occurs only in the Anatolian region, and the fourth is distributed in both in the Anatolian and Black Sea Regions. The TCS analysis corroborated the five distinct ML groups, but the ABGD analysis sorted one of the haplotypes (*P. major*-27) into an additional (sixth) group. This could be resolved with additional sampling, or could be attributed to the sensitivity of ABGD to the variation in the number of specimens analyzed in each species [32, 51]. As the biomedical importance of *P. major* (*s.l.*) is well documented [52–54], the robust discrimination between its component members is crucial for vector incrimination studies. The morphological characters were shown to be insufficient for a specific delimitation [12, 50] but high interspecific genetic distances between the five lineages distributed in Turkey (4.1–13.0%) suggests that *cox*1 barcoding is a useful method to discriminate between the members of the ‘Major Group’. Further comparative analysis of the additional nuclear markers and ecological data is necessary to clarify the taxonomic status of its sympatric members.

*Phlebotomus* (*Larroussius*) *kandelakii* is the proven vector of *L. infantum* in Georgia [55] and its potential role in VL transmission has been implicated in other several countries (reviewed in [40] and [56]), including Turkey [6]. *Phlebotomus kandelakii* (*s.l.*) comprises two described taxa: *Phlebotomus burneyi* is thought to be restricted to Pakistan, while *P. kandelakii* is found from Caucasia to the Middle East, and from Afghanistan to eastern Europe [57] and both occur in sympatry in Turkey [49, 58]. Members of *P. kandelakii* (*s.l.*) were collected in all three biogeographical regions of Turkey and the 52 *cox*1 sequences (20 haplotypes) formed three distinct groups by ABGD, TCS and ML analysis. The first two lineages comprised only haplotypes from the Anatolian populations, while the third lineage represented specimens from both the Black Sea and the Mediterranean regions. The haplotypes in lineage 2 shared 98.76–99.29% sequence similarity to *P. kandelakii* from Azerbaijan (GenBank: KY564178, KY564179 and KY564184). The purported differentiating male morpho-characters (e.g. paramere structure and the number of ascoids on certain antennal segments) are difficult to observe in these taxa, and females are isomorphic [9, 26], thus we were not able to assign species names with confidence to any of the three *P. kandelakii* lineages at this stage. Evaluation of the DNA sequences of *P. burneyi* and *P. kandelakii* from the type-localities in Georgia and Pakistan, respectively, is needed to concretely verify the identity and the taxonomic status of these taxa with respect to those found in Turkey.

Although the vectorial role of species in the subgenus *Adlerius* in *Leishmania* transmission has long been suspected, only a few species have been incriminated as leishmaniasis vectors. This is partly due to the inherent problems regarding the discrimination of the isomorphic females. Species identification therefore relies on male morphology, but the sympatric occurrence of different species complicates the vector incrimination studies. Of the 20 described sand fly species in the subgenus *Adlerius*, only five are reported from Turkey, none of which are regarded as biomedically important. *Phlebotomus simici* is a suspected vector of VL across its range (Middle East, eastern Mediterranean countries and Caucasia) [59, 60]. All 37 *P. simici* specimens collected from the Anatolian and the Mediterranean regions formed in a single cluster by all species delimitation methods. To our knowledge, there are no published data regarding the intraspecific variation of *P. simici*, but our results indicated that this widespread species is represented by a single lineage in Turkey, with a low intraspecific K2P distance (1.1%). *Phlebotomus brevis*, another suspected vector of *L. infantum*, has been reported from Greece, Malta, Turkey, Iran and Caucasia. In concordance with the previous records from Turkey [3, 49], *P. brevis* was notably rare in our collections. Resultant barcodes from our three specimens from the westernmost and southwestern localities of the Mediterranean region clustered tightly, and were clearly separated from their consubgeners. Similarly, *P. balcanicus* and *P. kyreniae* were rare, and restricted to only specific locations in the Mediterranean and in the Anatolian regions, respectively. *Phlebotomus balcanicus* is the proven vector of VL in Georgia and *P. kyreniae* is a suspected vector of *L. infantum* in Cyprus. Despite their morphological resemblance, these two species were grouped in independent clusters and appeared as sister taxa in the ML tree of the subgenus *Adlerius*. Widely distributed in Eurasia (Middle East, Caucasia and Turkey), *P. halepensis* is a suspected vector of leishmaniasis and has been experimentally indicated as a competent species for both types of the disease [61]. All methods identified a single group for the *P. halepensis* specimens (*n* = 34) from the Anatolian and the Black Sea regions, and the ML analysis revealed a single lineage for the 20 haplotypes analyzed without any sign of significant population structuring (intraspecific K2P distance = 1.0%). Importantly, we were able to assign most of the isomorphic *Adlerius* female specimens to species, irrespective of their geographical origin. Neither the sympatric occurrence of different species, nor the geographical distance between the sampling locations of the same species, influenced the specific discrimination between the females and we were able to provide the barcode sequences for each of the *Adlerius* taxa distributed in Turkey, based on female specimens. However, the identity of the lineage including seven *Adlerius* haplotypes from the Anatolian region which clustered as the sister species of *P. brevis* from the Mediterranean region, remain unresolved.

Five Turkish species belong to the subgenus *Transphlebotomus*, but their vectorial status remains unknown. *Phlebotomus mascittii* is widely distributed in Mediterranean countries [49, 62, 63], stretching into Central Europe [64–66] as far north as Slovakia [67]. Recently, *P. mascittii* was verified in Serbia [68] and Slovenia [69] using *cox*1 barcodes and other DNA markers. Despite targeted collections in Anatolian and Mediterranean provinces where *P. mascittii* was previously recorded [3, 49], the species was not detected, and the prior records of *P. mascittii* in Turkey remains questionable. The distributions of the other *Transphlebotomus* species appear more focused: *P. canaaniticus* is found in the Middle East [70–72], and *P. economidesi*, previously reported only from Cyprus [73], was recently described in sympatry with *P. anatolicus* and *P. killicki* in Turkey [18]. Herein, *cox*1 barcodes of two specimens collected from the Mediterranean region were molecularly confirmed as *P. anatolicus* and *P. killicki.* The first record of *P. killicki* in Cyprus was also verified based on *cox*1 sequence correlation [20].

The role of *Sergentomyia* spp. in the transmission of mammalian diseases is debatable [74], but they are recognized as the vectors of *Sauraleishmania* parasites that affect reptiles [75]. *Sergentomyia antennata*, *S. dentata*, *S. minuta* and *S. theodori* are reported from Turkey, and *Sauroleishmania* spp. DNA was detected in *S*. *dentata* in southwestern Turkey [4, 45]. All *Sergentomyia* specimens collected during this study were identified as *S. dentata* or *S. minuta* by morphology. Two *cox*1 barcode clusters were determined in the 26 *S. dentata* haplotypes from the Mediterranean region that closely correlate to the geographical origin of the specimens: one from populations in the western part of the Mediterranean region, and the other from the southern localities of the same region. With the exception of one specimen from the Anatolian region, all *S. minuta* specimens barcoded were from the Mediterranean region. Regardless of the geographical locations of the sampling stations, three barcode clusters were generated for the 28 *S. minuta* haplotypes we analyzed with an interspecific genetic distance range of 3.0–4.1%. Deep mitochondrial intraspecific variation in *S. minuta* has been recently reported. Analysis of concatenated mtDNA *cytb*-*cox*1 sequences of *S. minuta* from across Europe and North Africa determined four distinct lineages, irrespective of their geographical origins [76]. Conversely, *S. minuta* specimens from Crete, Cyprus and mainland Greece clustered in three distinct lineages by locality [20]. Distributed across all three continents of the Old World, *S. minuta* exhibits morphologically diverse populations. Whether these populations represent different subspecies is controversial (see review in [76]). It is evident that *S. minuta* is not a molecularly or morphologically homogenous species, but its true taxonomic status is likely only to be clarified following molecular analysis of specimens across its entire distributional range.

Using the *cox*1 barcode sequences, 33 distinct genetic clusters (MOTUs) of sand flies were identified, increasing the documented number of Turkish species based in taxonomic approaches by at least five species. Non-parametric richness estimator (Chao2) suggested the existence of two additional species, which may correspond to *S. antennata* and *S. theodori* that we did not detect during our survey, but that have previously been reported in the country. Sand fly biodiversity appears highest in the Anatolian region (26 MOTUs), followed by the Mediterranean region (23 MOTUs), and then the Black Sea region (17 MOTUs). As indicated by Chao2, we did not recover the expected species richness either in the Anatolian or Black Sea regions, but captured the diversity of the Mediterranean sand fly fauna appropriately. It is evident that including more sampling locations will be helpful to discover the undetected species/MOTUs present in these regions.

## Conclusions

The comprehensive DNA barcode reference library of Turkish sand fly diversity generated in this study will greatly contribute to the accurate identification of sand flies in entomological, ecological and epidemiological studies in Turkey and beyond. In addition, this study presents novel distribution data of proven or probable sand fly vectors in the Black Sea region for the first time. The extensive distribution of *P. tobbi* and *P. sergenti* (*s.l.*), the proven vectors of leishmaniasis, along with other medically important *Larroussius* taxa in Turkey, highlights the importance of ongoing surveillance studies to evaluate the sand fly-borne disease risk in previously overlooked parts of the country. The discriminatory power of DNA barcoding for the robust identification of the morphologically unidentifiable specimens, e.g. *Adlerius* females, is invaluable in vector incrimination studies. Our results confirm the utility of *cox*1 DNA barcoding for the robust identification of sand flies, and for revealing cryptic genetic diversity within these taxa. Future multi-locus DNA studies integrated with ecological and behavioral criteria is now crucial for delimiting species boundaries in genetically diverse taxa, especially where introgression events are possible.

## Additional files

**Additional file 1: Table S1.** Information regarding the sand fly collection sites and the number of traps used in each locality.

**Additional file 2: Figure S1.** Neighbor Joining (NJ) tree using the 741 *cox*1 sequences obtained for Turkish sand fly specimens. The bootstrap values higher than 50% are indicated.

**Additional file 3: Figure S2.** Haplotype network obtained for the 56 *P. papatasi* specimens analyzed from Turkey. Haplotypes are sized according to their relative frequencies and colored by their geographical origin. Missing haplotypes are denoted by small black circles and the numbers of mutational steps are represented by the dashes.

**Additional file 4: Figure S3.** Haplotype networks obtained for the 83 *Paraphlebotomus* specimens analyzed from Turkey (a *P. alexandri*, b *P. sergenti* (*s.l.*), c *P. caucasicus*, *P. jacusieli* and *Paraphlebotomus* sp.). Haplotypes are sized according to their relative frequencies and colored by their geographical origin. Missing haplotypes are denoted by small black circles and the numbers of mutational steps are represented by the dashes.

**Additional file 5: Table S2.** Interspecific and intraspecific (diagonal line) *cox*1 sequence divergence based on K2P model obtained for Turkish sand flies.

**Additional file 6: Figure S4.** Haplotype network obtained for the 107 *P. tobbi* specimens analyzed from Turkey. Haplotypes are sized according to their relative frequencies and colored by their geographical origin. Missing haplotypes are denoted by small black circles and the numbers of mutational steps are represented by the dashes.

**Additional file 7: Figure S5.** Haplotype network obtained for the 96 *P. perfiliewi* (*s.l.*) specimens analyzed from Turkey. Haplotypes are sized according to their relative frequencies and colored by their geographical origin. Missing haplotypes are denoted by small black circles and the numbers of mutational steps are represented by the dashes.

**Additional file 8: Figure S6.** Haplotype network obtained for the 169 *P. major* (*s.l.*) specimens analyzed from Turkey. Haplotypes are sized according to their relative frequencies and colored by their geographical origin. Missing haplotypes are denoted by small black circles and the numbers of mutational steps are represented by the dashes.

**Additional file 9: Figure S7.** Haplotype network obtained for the 52 *P. kandelakii* (*s.l.*) specimens analyzed from Turkey. Haplotypes are sized according to their relative frequencies and colored by their geographical origin. Missing haplotypes are denoted by small black circles and the numbers of mutational steps are represented by the dashes.

**Additional file 10: Figure S8.** Haplotype networks obtained for the 101 *Adlerius* specimens analyzed from Turkey (a *P. simici*; b *P. brevis* and *Adlerius* sp.; c *P. balcanicus*, *P. halepensis* and *P. kyreniae*). Haplotypes are sized according to their relative frequencies and colored by their geographical origin. Missing haplotypes are denoted by small black circles and the numbers of mutational steps are represented by the dashes.

**Additional file 11: Figure S9.** Haplotype networks obtained for the 16 *Transphlebotomus* specimens analyzed from Turkey. Haplotypes are sized according to their relative frequencies and colored by their geographical origin. Missing haplotypes are denoted by small black circles and the numbers of mutational steps are represented by the dashes.

**Additional file 12: Figure S10.** Haplotype networks obtained for the 62 *Sergentomyia* specimens analyzed from Turkey (a *S. dentata*; b *S. minuta*). Haplotypes are sized according to their relative frequencies and colored by their geographical origin. Missing haplotypes are denoted by small black circles and the numbers of mutational steps are represented by the dashes.

**Additional file 13: Figure S11.** The distribution of the MOTUs observed in the three biogeographical regions of Turkey. Different colors represent the different sand fly species delineated by all three methods (ABGD, ML and TCS).

## Data Availability

Data supporting the conclusions of this article are included within the article and its additional files. The unique *cox*1 haplotypes obtained for all sand fly species from Turkey were deposited in GenBank under the Accession Numbers MN086366–MN086773.

## Abbreviations

ABGD: Automatic Barcode Gap Discovery program
CDC: Centre of Disease Control and Prevention
*cox*1: cytochrome *c* oxidase subunit 1
CL: cutaneous leishmaniasis
Ef-1α: elongation factor 1-alpha
K2P: Kimura’s two parameter
ML: Maximum Likelihood
MOTU: molecular operational taxonomic unit
NJ: Neighbor Joining
PCR: polymerase chain reaction
VL: visceral leishmaniasis
