# Supplementary figures and images for: Revision of the species composition and distribution of Turkish sand flies using DNA barcodes
Source: Parasit Vectors. 2019 Aug 22;12:410. doi: 10.1186/s13071-019-3669-3 (PMC6704649; doi:10.1186/s13071-019-3669-3)

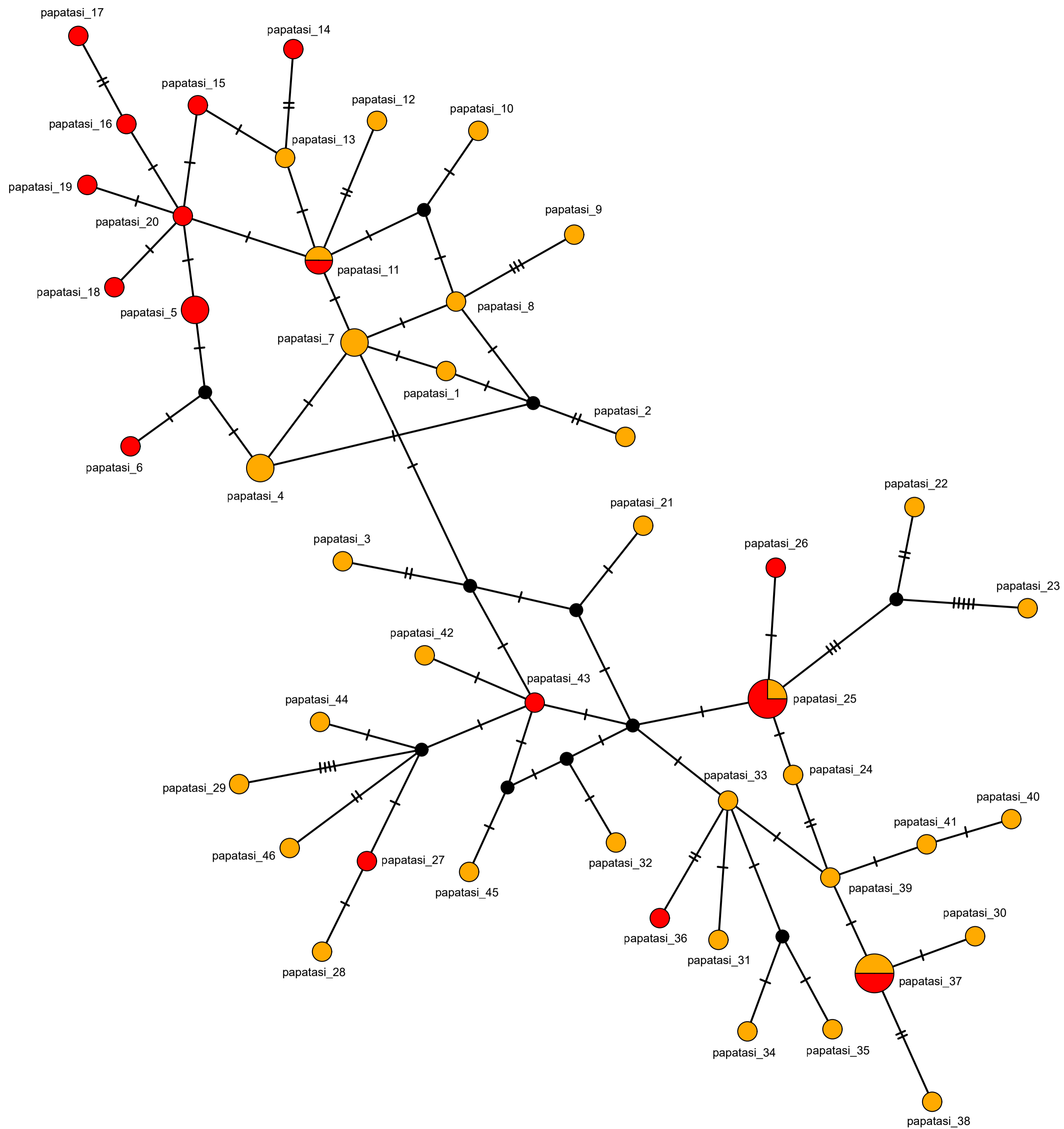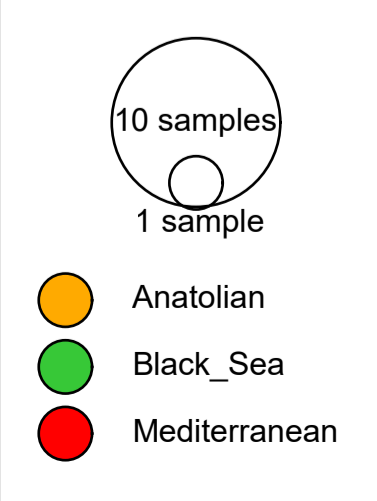

Supplement: Supplementary file 3 — Additional file 3: Figure S2. Haplotype network obtained for the 56 P. papatasi specimens analyzed from Turkey. Haplotypes are sized according to their relative frequencies and colored by their geographical origin. Missing haplotypes are denoted by small black circles and the numbers of mutational steps are represented by the dashes. [file 13071_2019_3669_MOESM3_ESM.pdf]

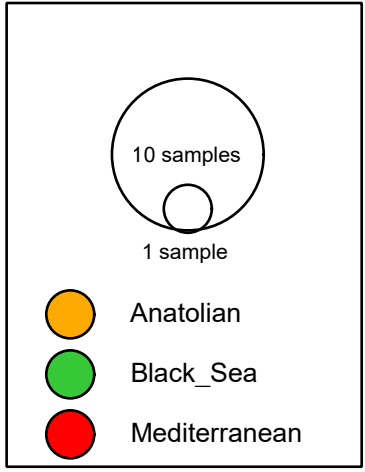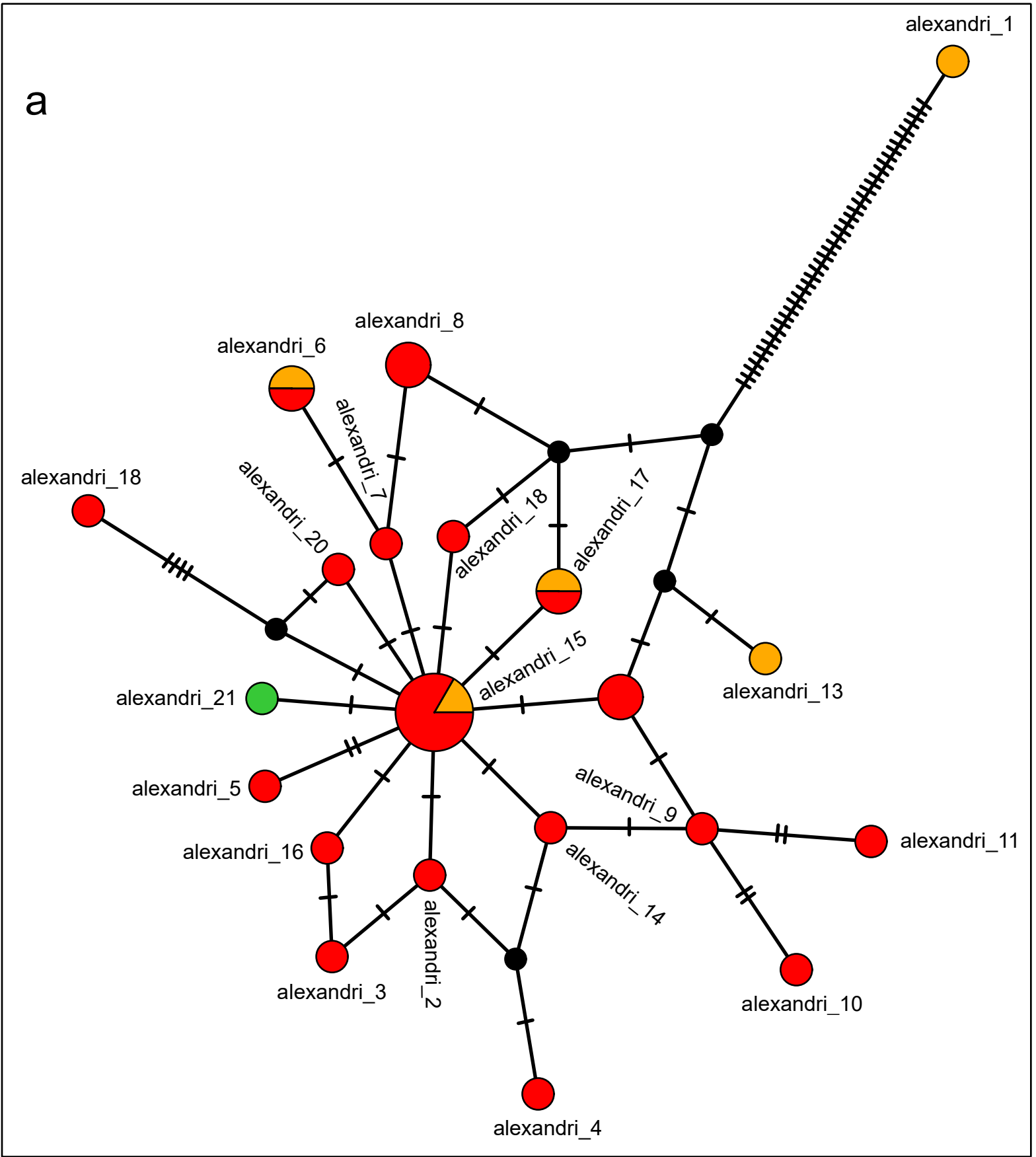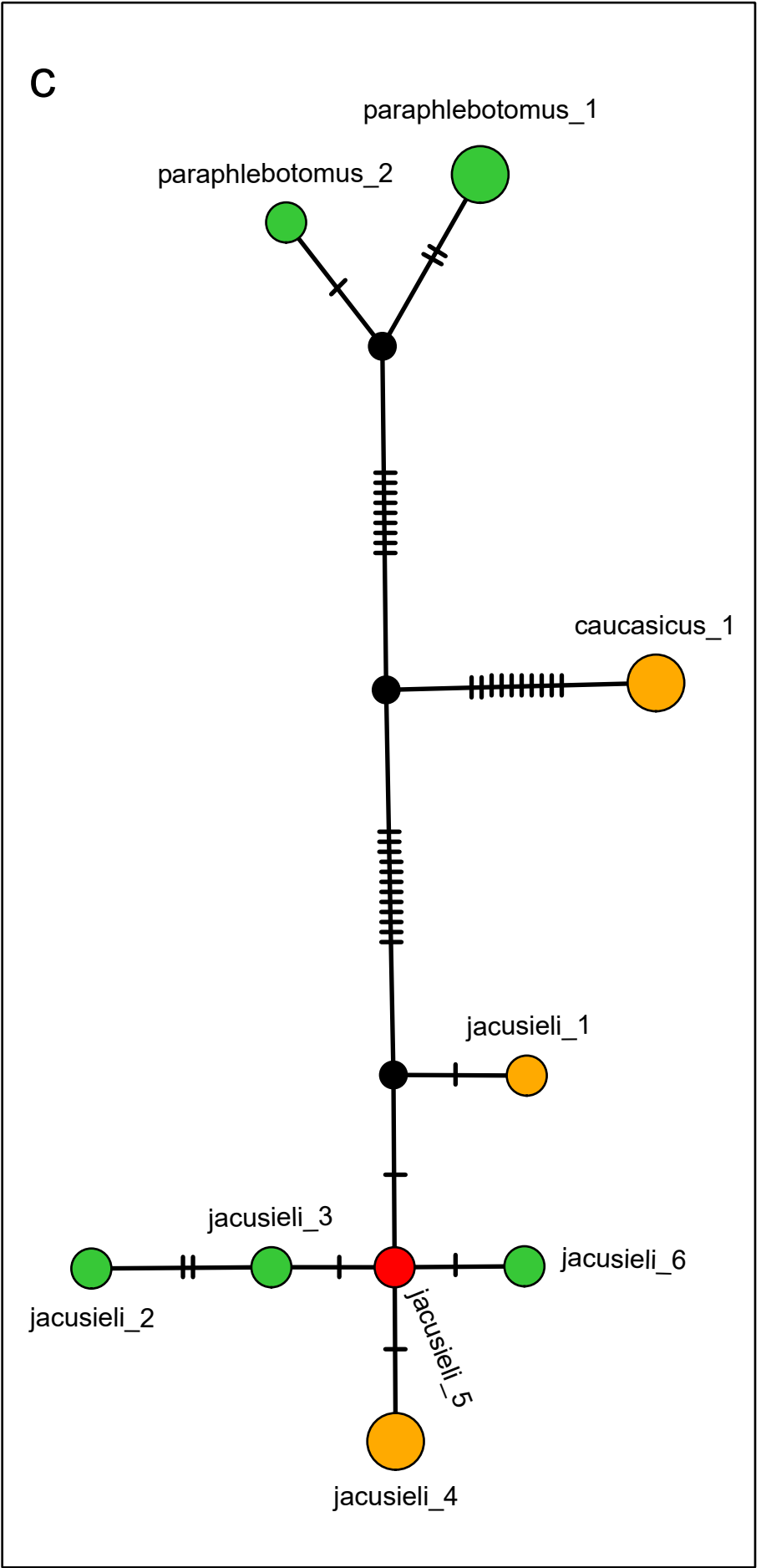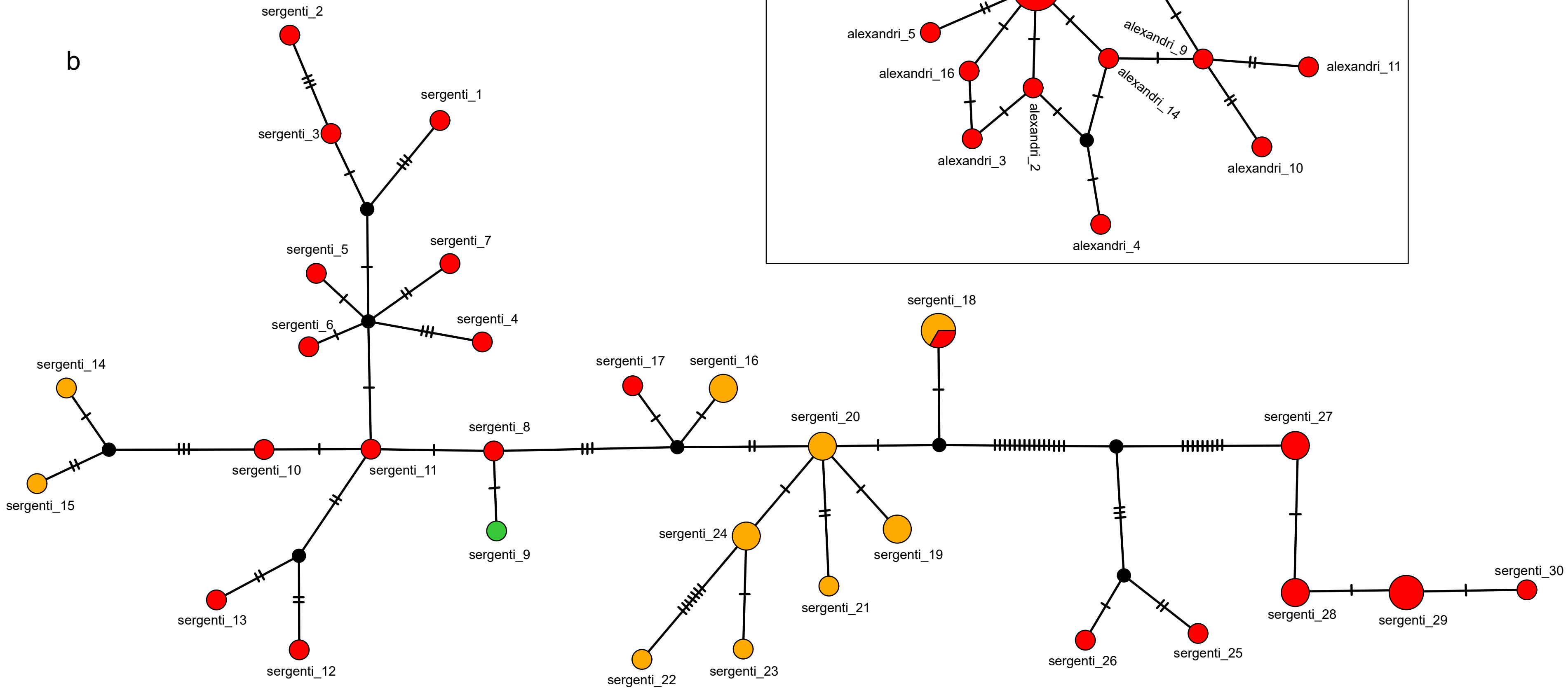

Supplement: Supplementary file 4 — Additional file 4: Figure S3. Haplotype networks obtained for the 83 Paraphlebotomus specimens analyzed from Turkey (a P. alexandri, b P. sergenti (s.l.), c P. caucasicus, P. jacusieli and Paraphlebotomus sp.). Haplotypes are sized according to their relative frequencies and colored by their geographical origin. Missing haplotypes are denoted by small black circles and the numbers of mutational steps are represented by the dashes. [file 13071_2019_3669_MOESM4_ESM.pdf]

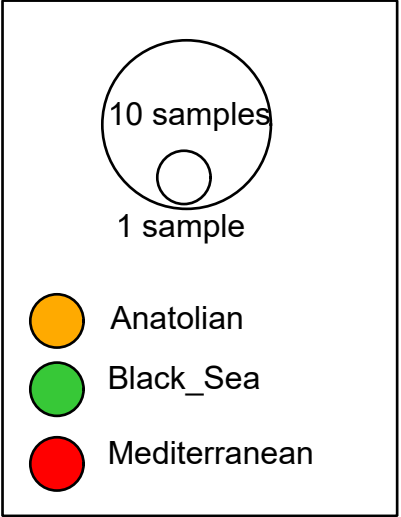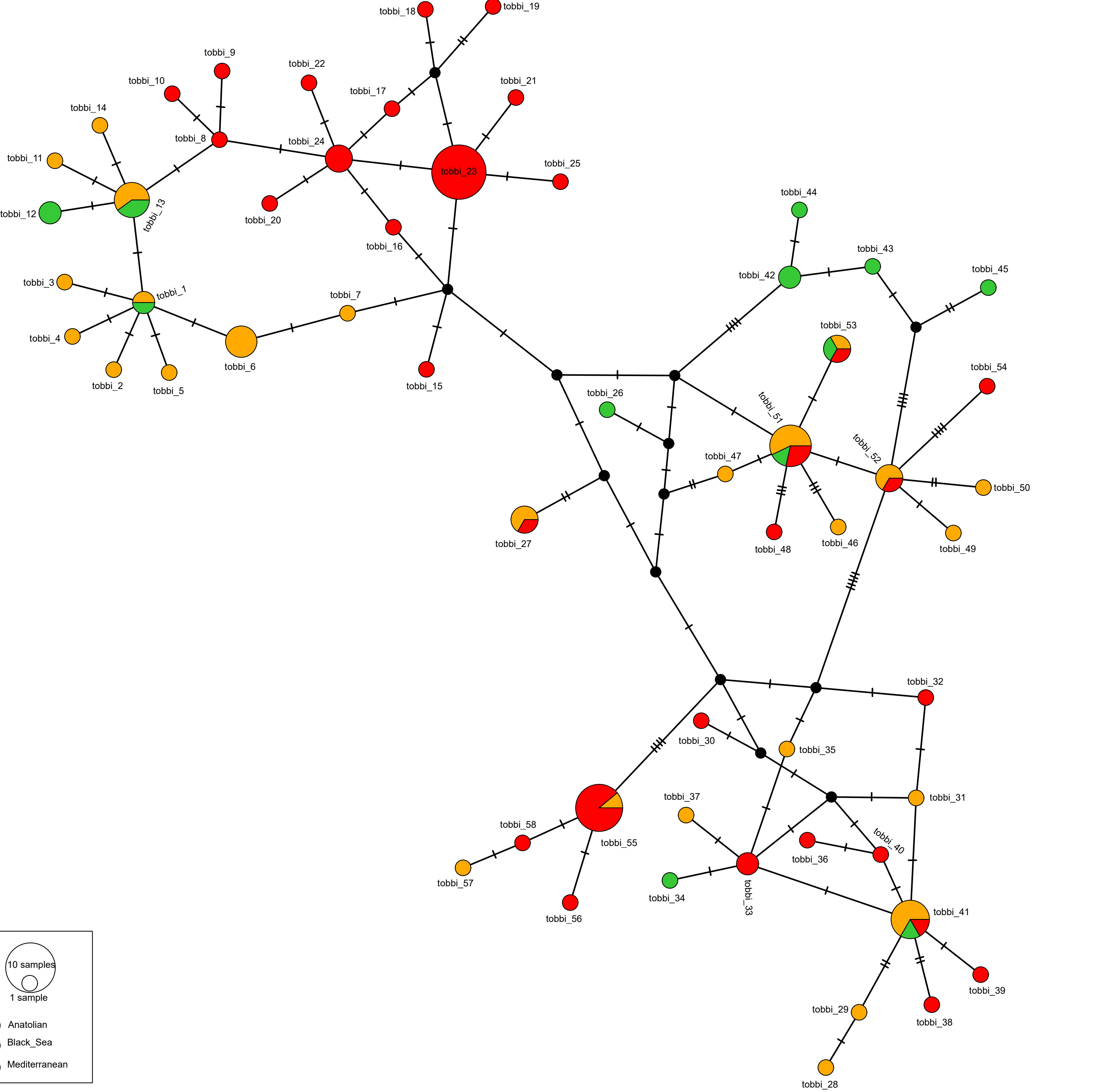

Supplement: Supplementary file 6 — Additional file 6: Figure S4. Haplotype network obtained for the 107 P. tobbi specimens analyzed from Turkey. Haplotypes are sized according to their relative frequencies and colored by their geographical origin. Missing haplotypes are denoted by small black circles and the numbers of mutational steps are represented by the dashes. [file 13071_2019_3669_MOESM6_ESM.pdf]

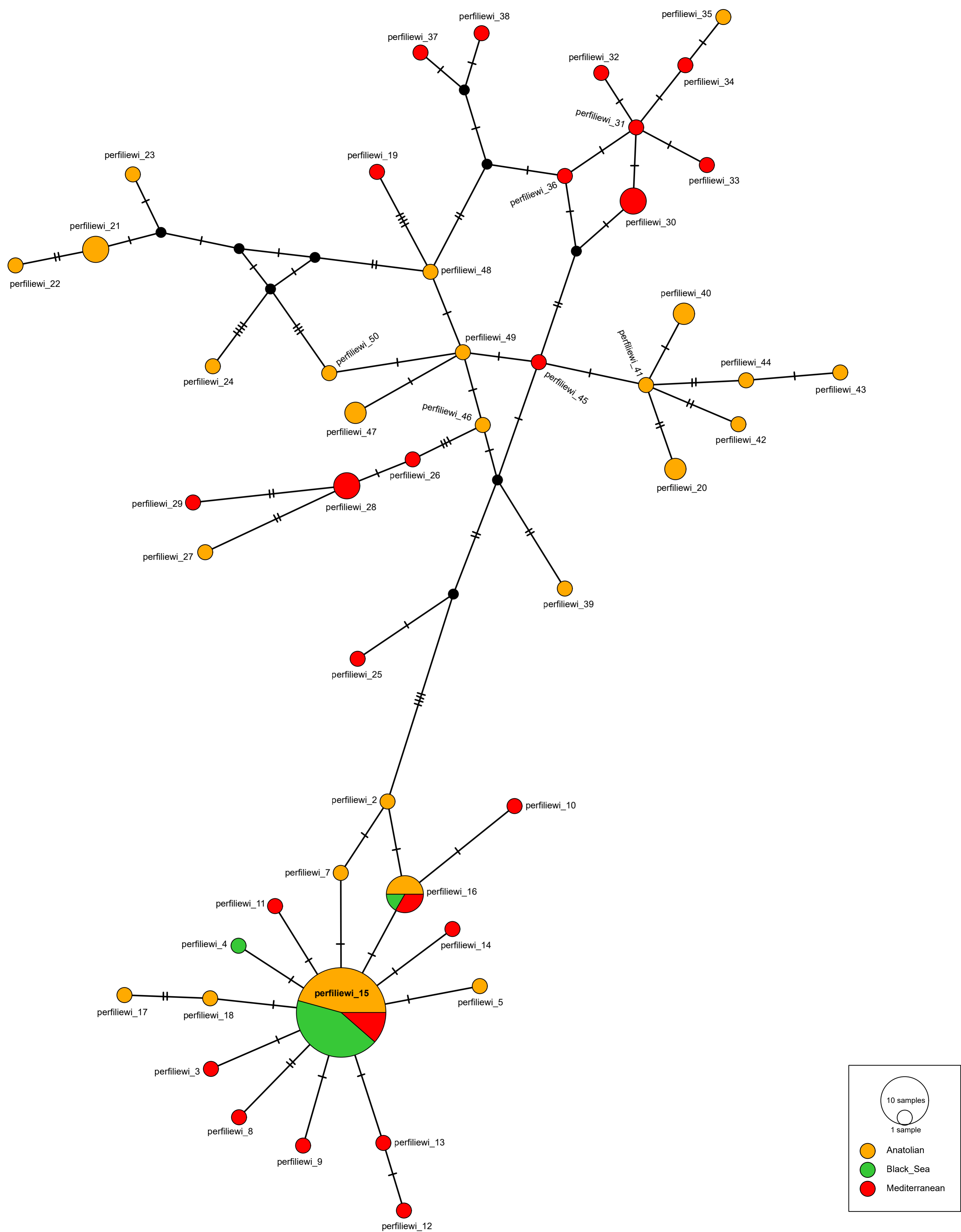

Supplement: Supplementary file 7 — Additional file 7: Figure S5. Haplotype network obtained for the 96 P. perfiliewi (s.l.) specimens analyzed from Turkey. Haplotypes are sized according to their relative frequencies and colored by their geographical origin. Missing haplotypes are denoted by small black circles and the numbers of mutational steps are represented by the dashes. [file 13071_2019_3669_MOESM7_ESM.pdf]

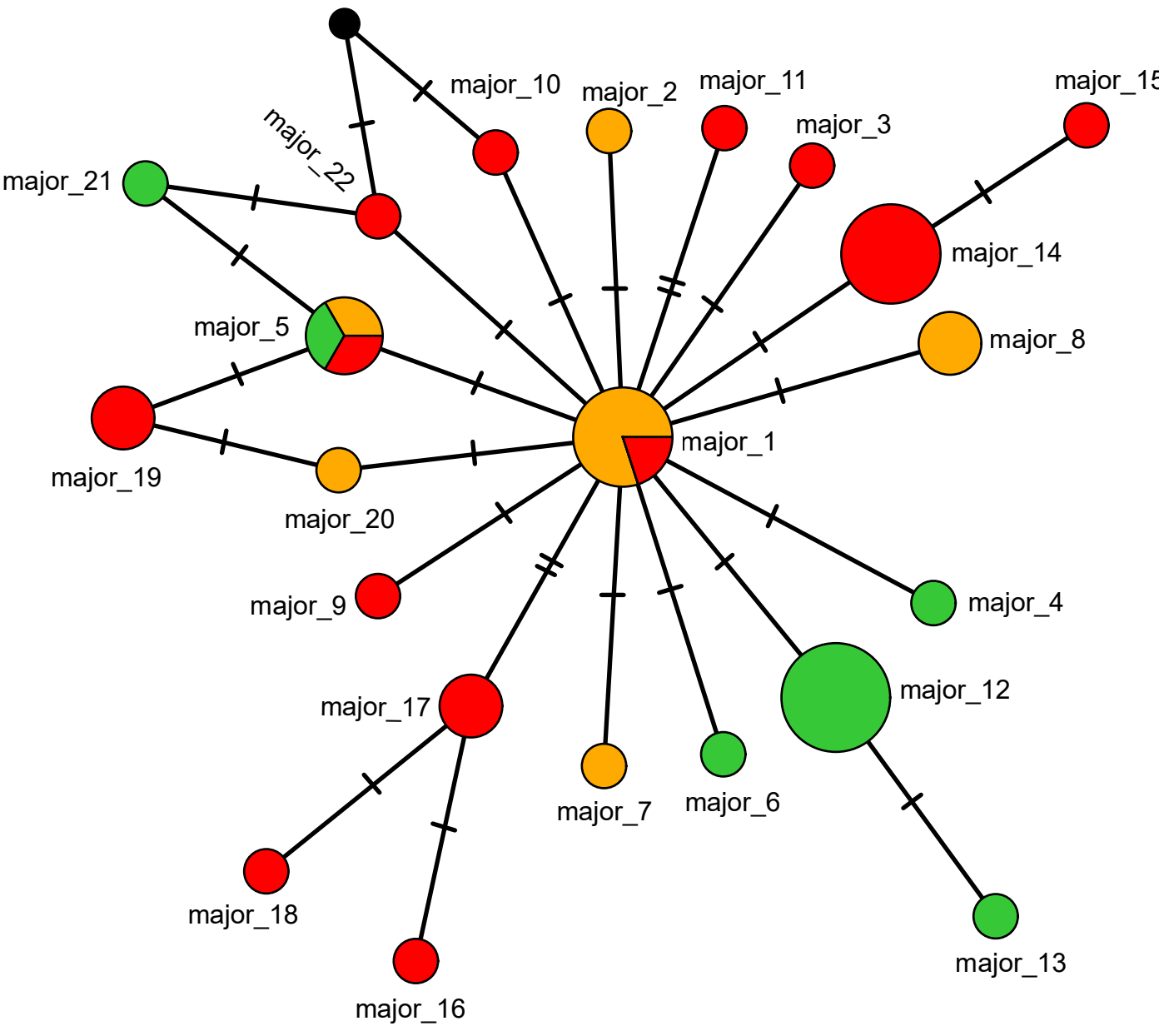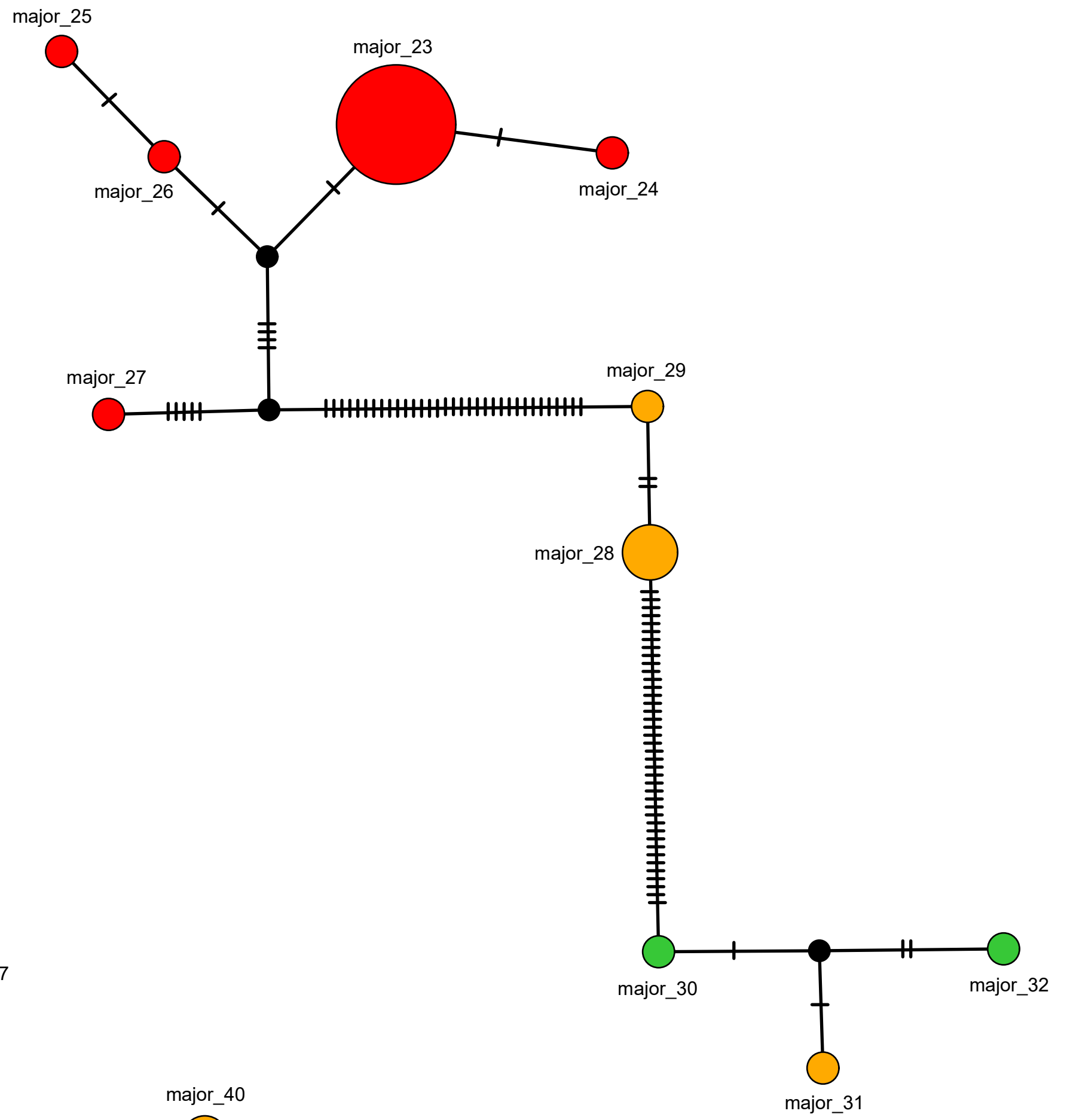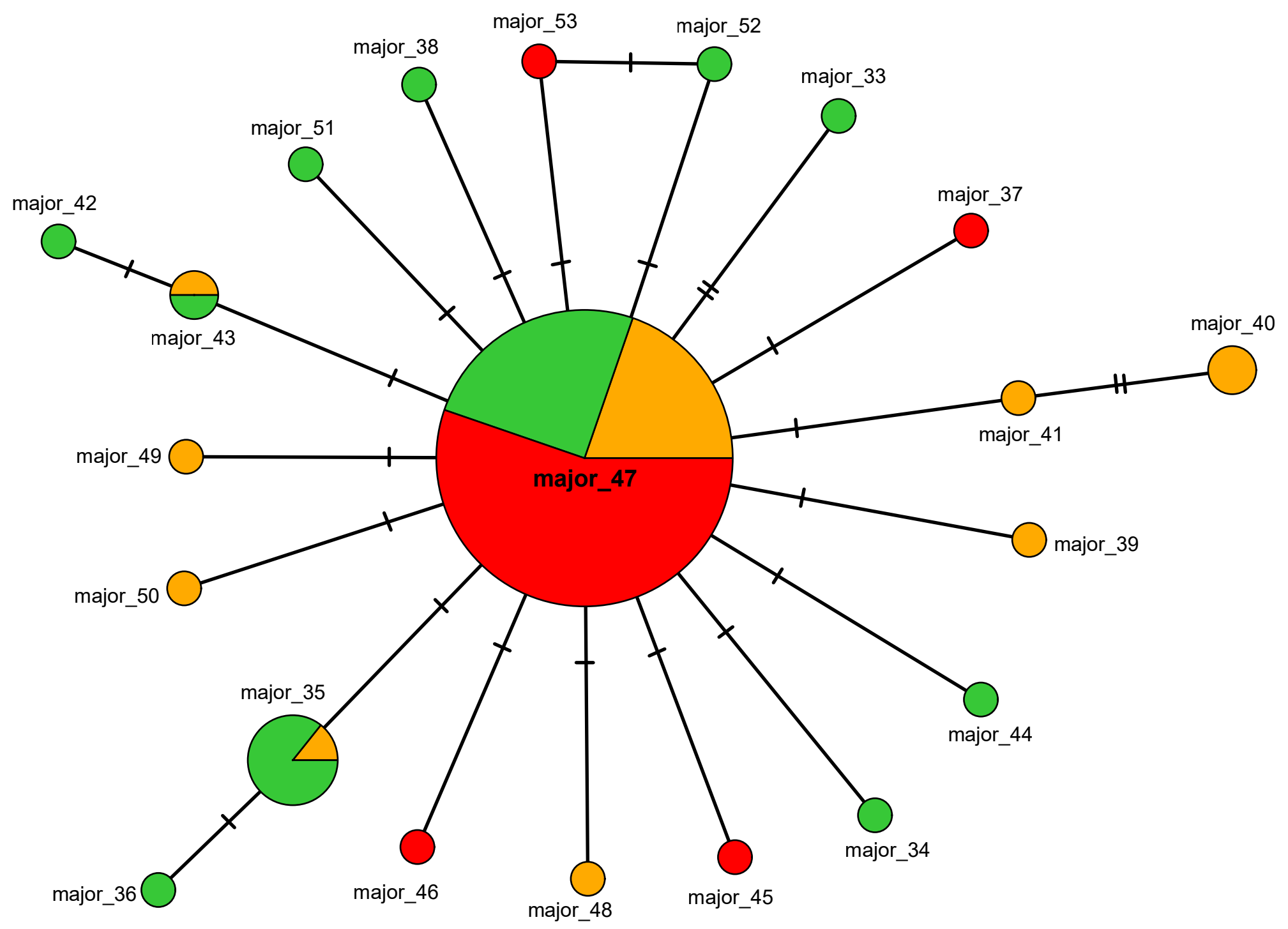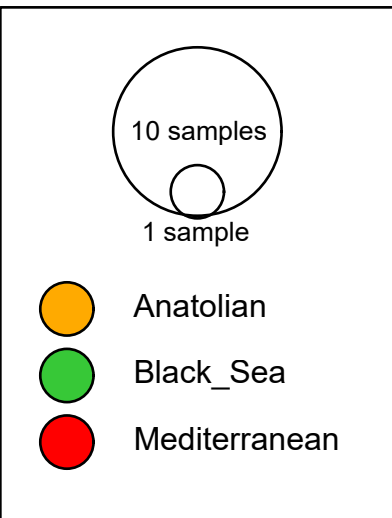

Supplement: Supplementary file 8 — Additional file 8: Figure S6. Haplotype network obtained for the 169 P. major (s.l.) specimens analyzed from Turkey. Haplotypes are sized according to their relative frequencies and colored by their geographical origin. Missing haplotypes are denoted by small black circles and the numbers of mutational steps are represented by the dashes. [file 13071_2019_3669_MOESM8_ESM.pdf]

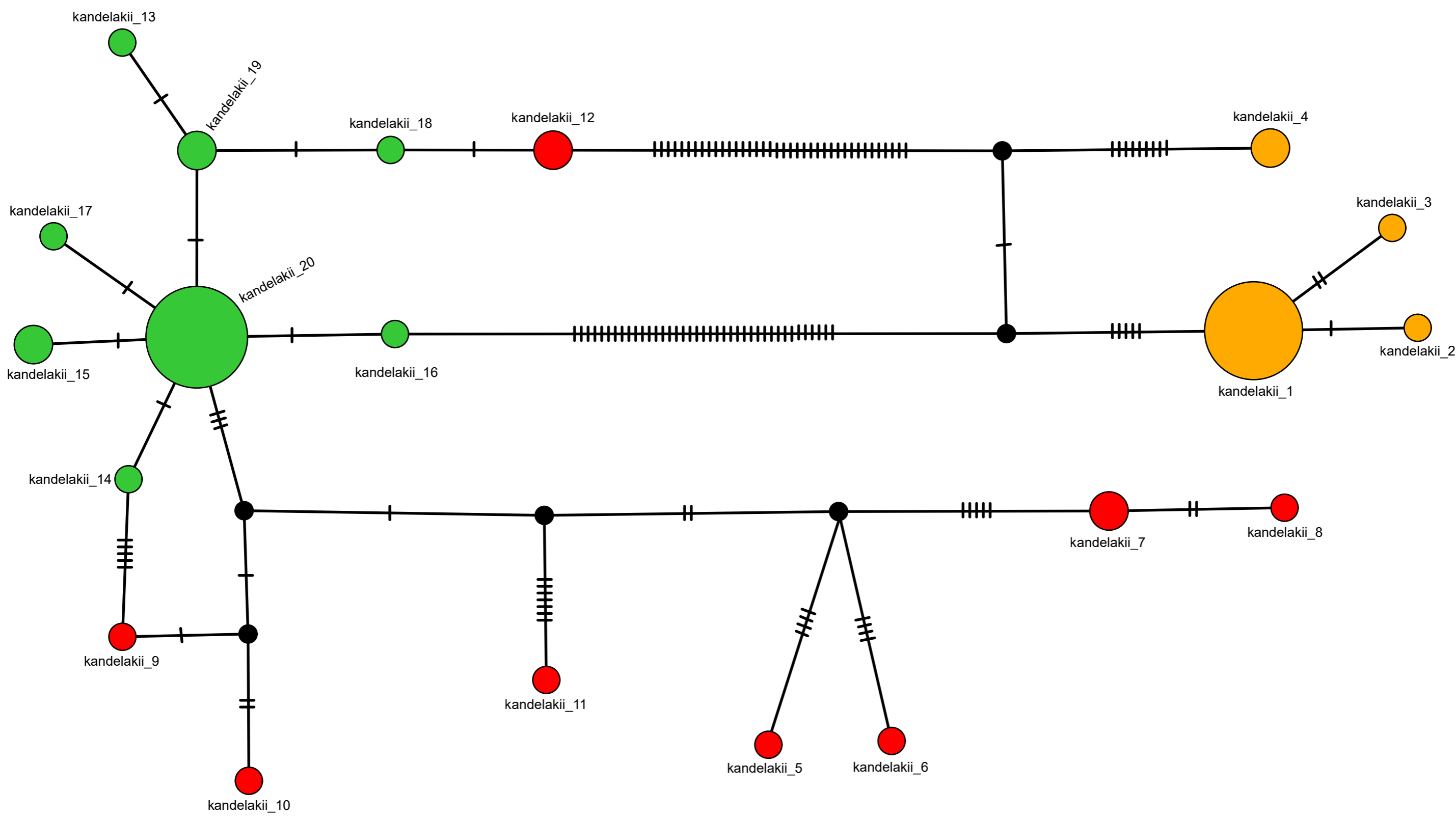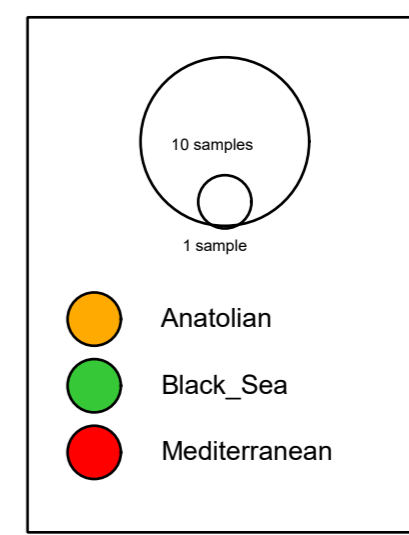

Supplement: Supplementary file 9 — Additional file 9: Figure S7. Haplotype network obtained for the 52 P. kandelakii (s.l.) specimens analyzed from Turkey. Haplotypes are sized according to their relative frequencies and colored by their geographical origin. Missing haplotypes are denoted by small black circles and the numbers of mutational steps are represented by the dashes. [file 13071_2019_3669_MOESM9_ESM.pdf]

a

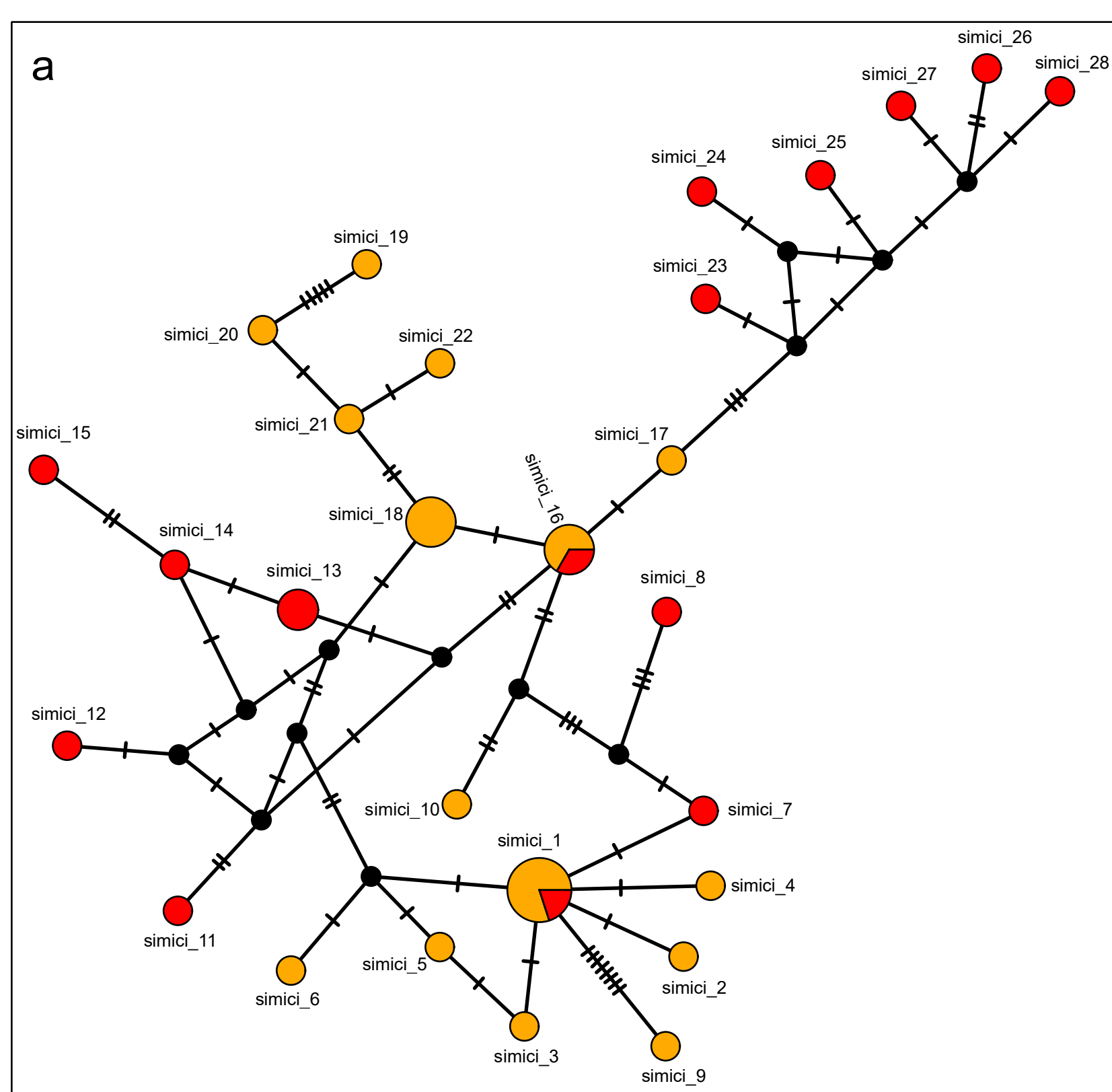

c

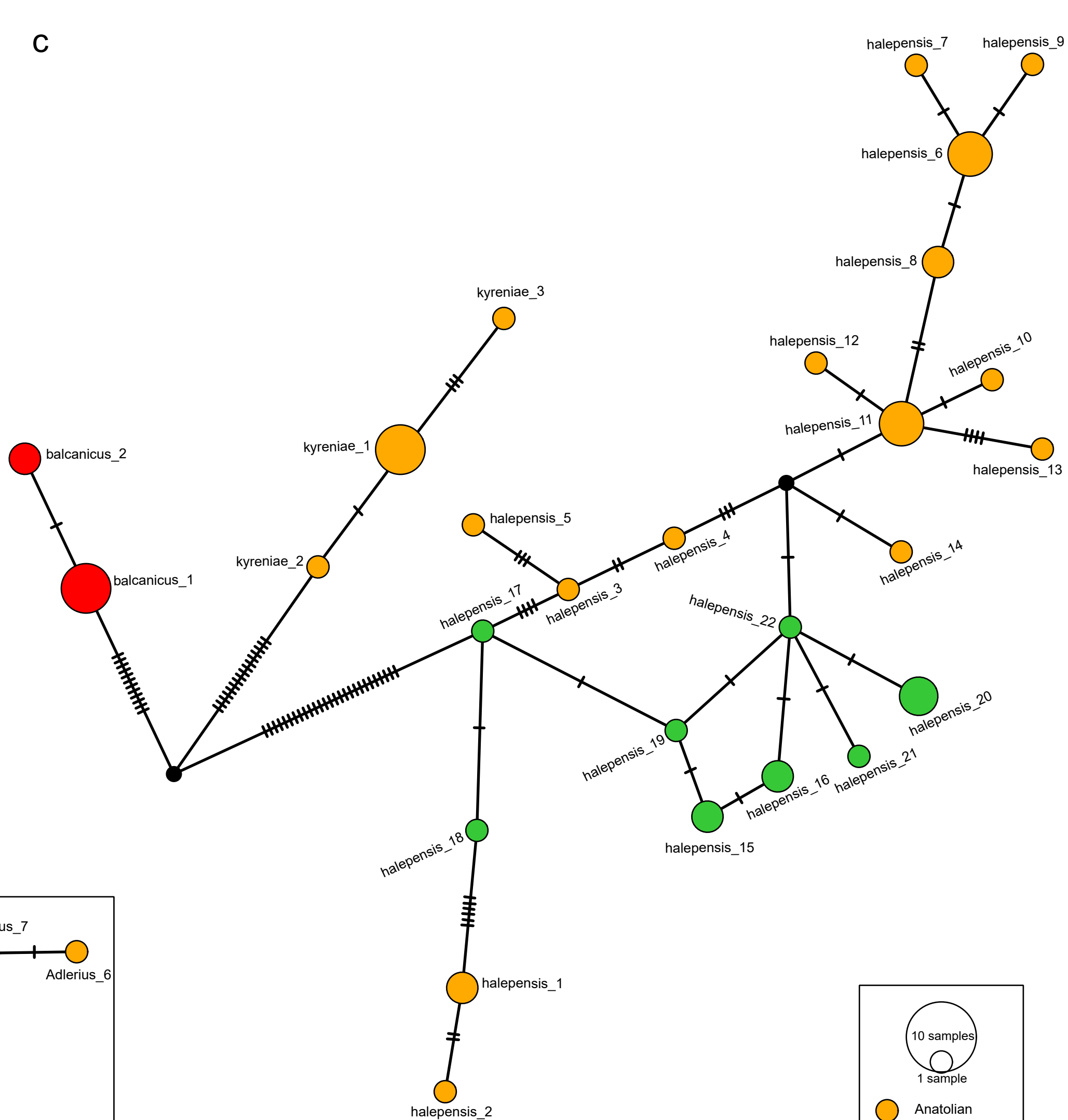

b

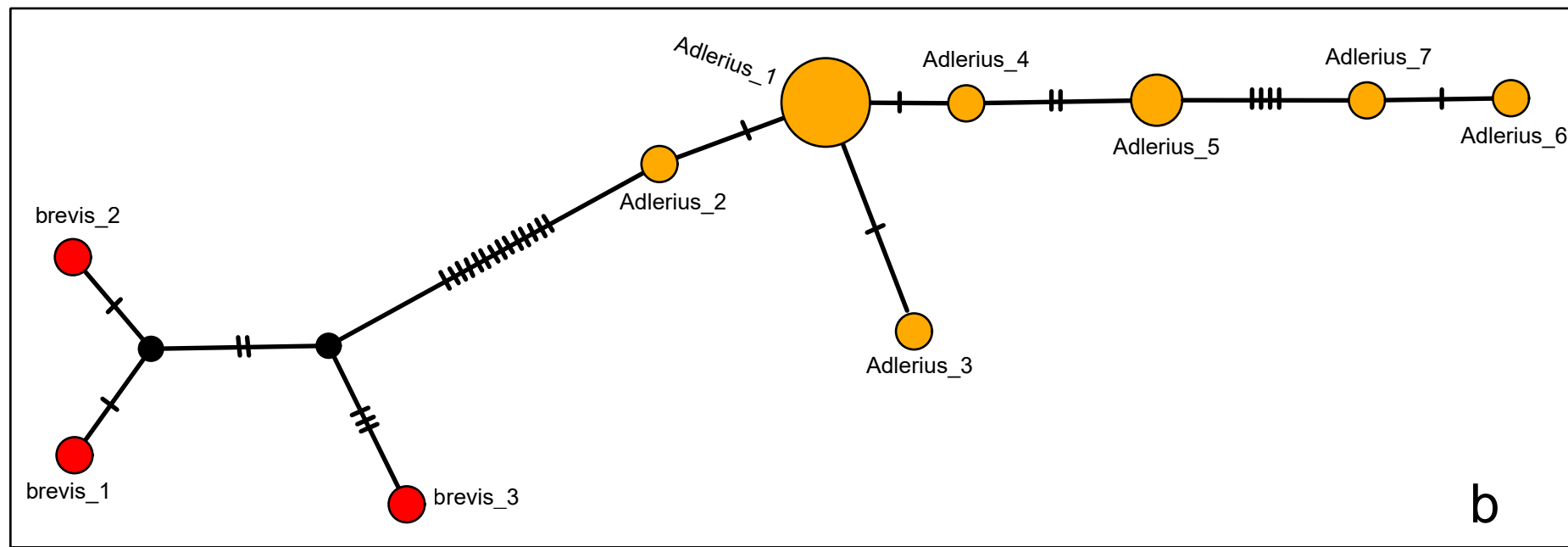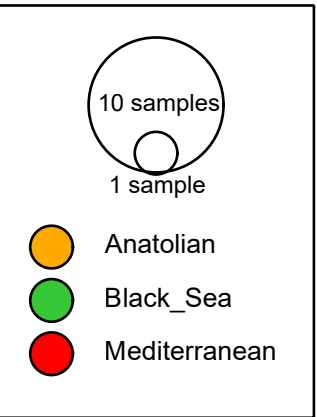

Supplement: Supplementary file 10 — Additional file 10: Figure S8. Haplotype networks obtained for the 101 Adlerius specimens analyzed from Turkey (a P. simici; b P. brevis and Adlerius sp.; c P. balcanicus, P. halepensis and P. kyreniae). Haplotypes are sized according to their relative frequencies and colored by their geographical origin. Missing haplotypes are denoted by small black circles and the numbers of mutational steps are represented by the dashes. [file 13071_2019_3669_MOESM10_ESM.pdf]

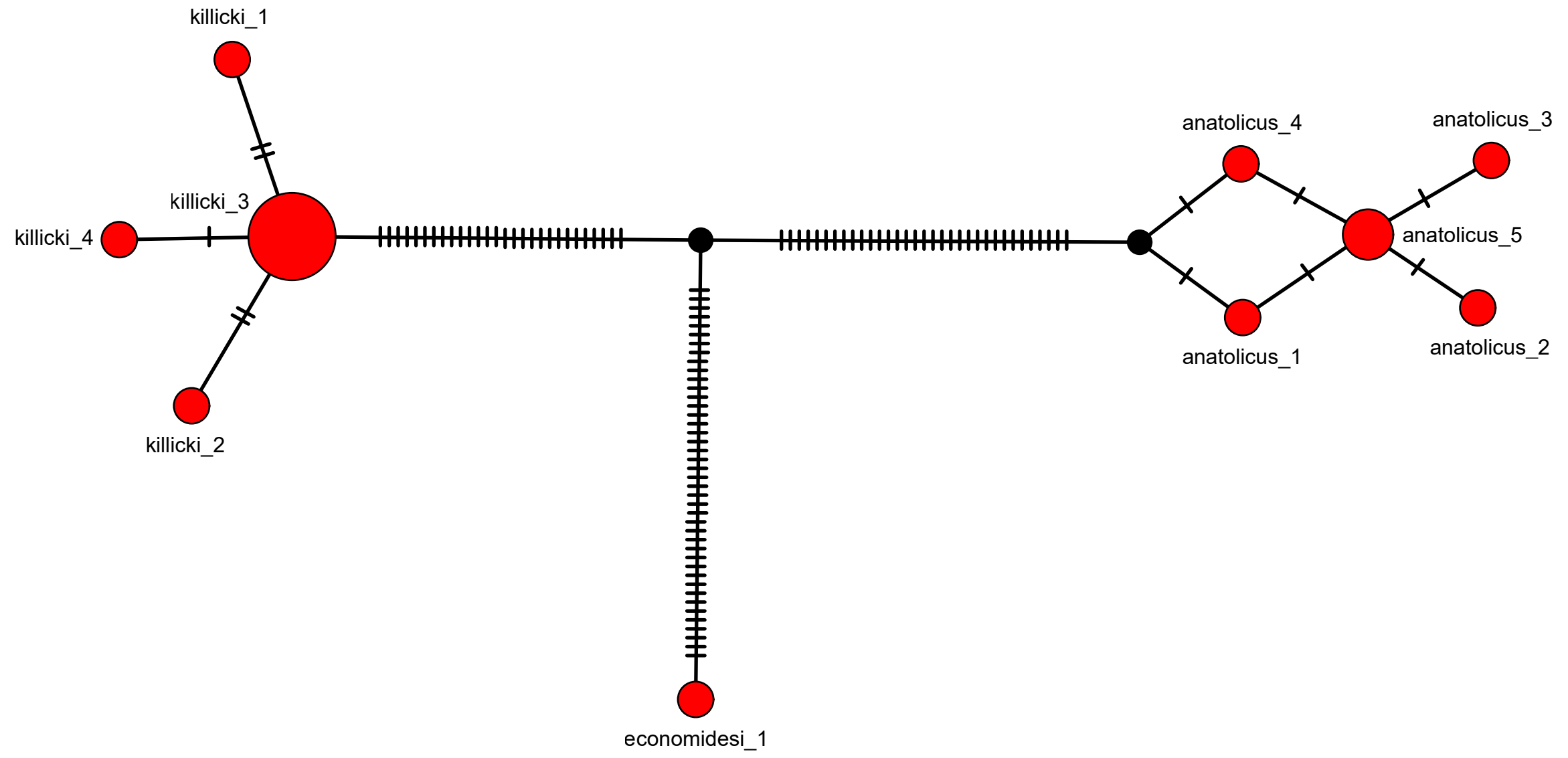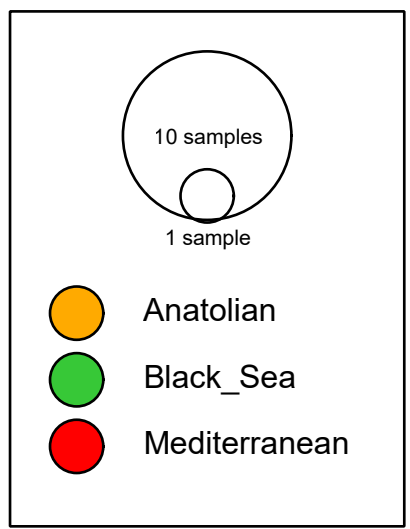

Supplement: Supplementary file 11 — Additional file 11: Figure S9. Haplotype networks obtained for the 16 Transphlebotomus specimens analyzed from Turkey. Haplotypes are sized according to their relative frequencies and colored by their geographical origin. Missing haplotypes are denoted by small black circles and the numbers of mutational steps are represented by the dashes. [file 13071_2019_3669_MOESM11_ESM.pdf]

a

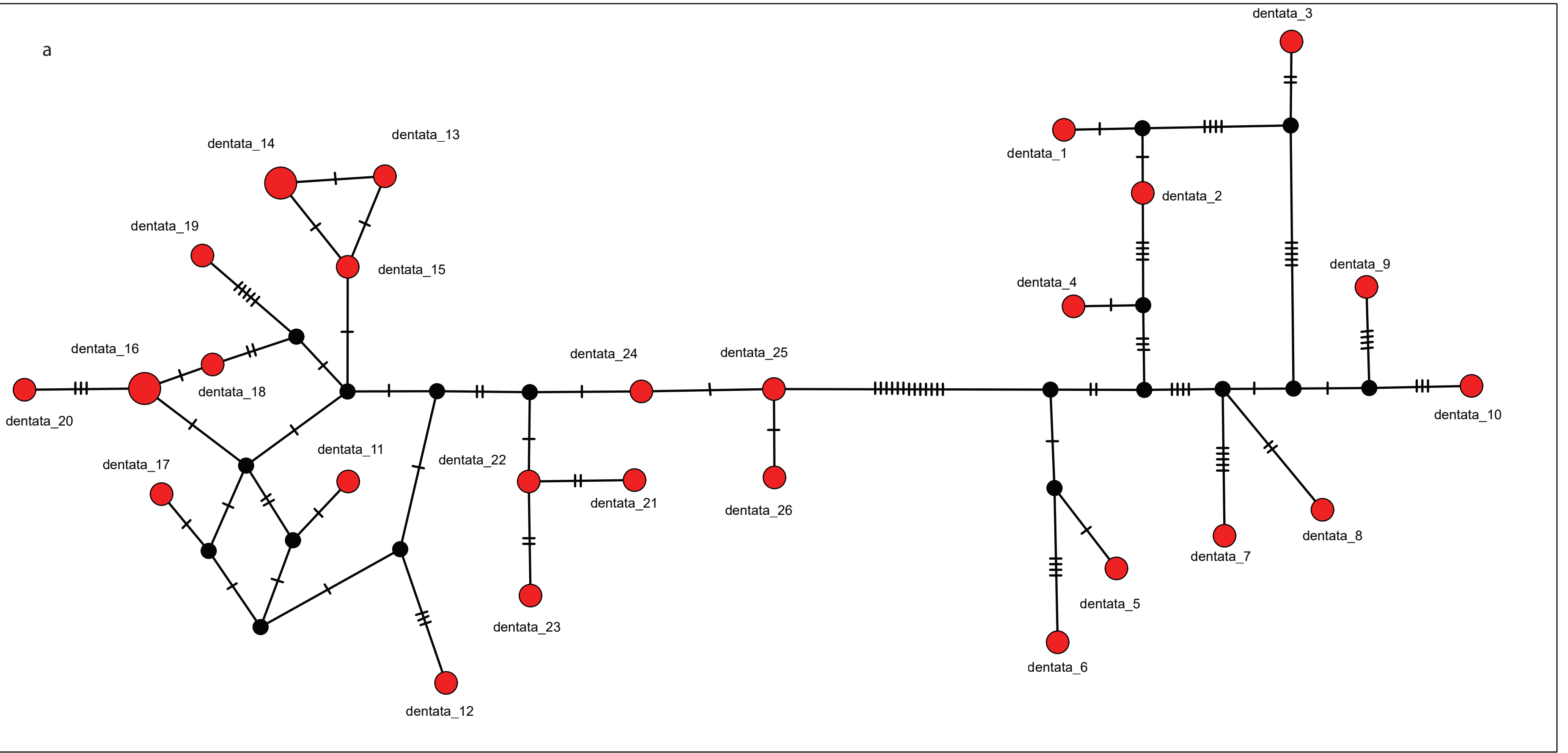

b

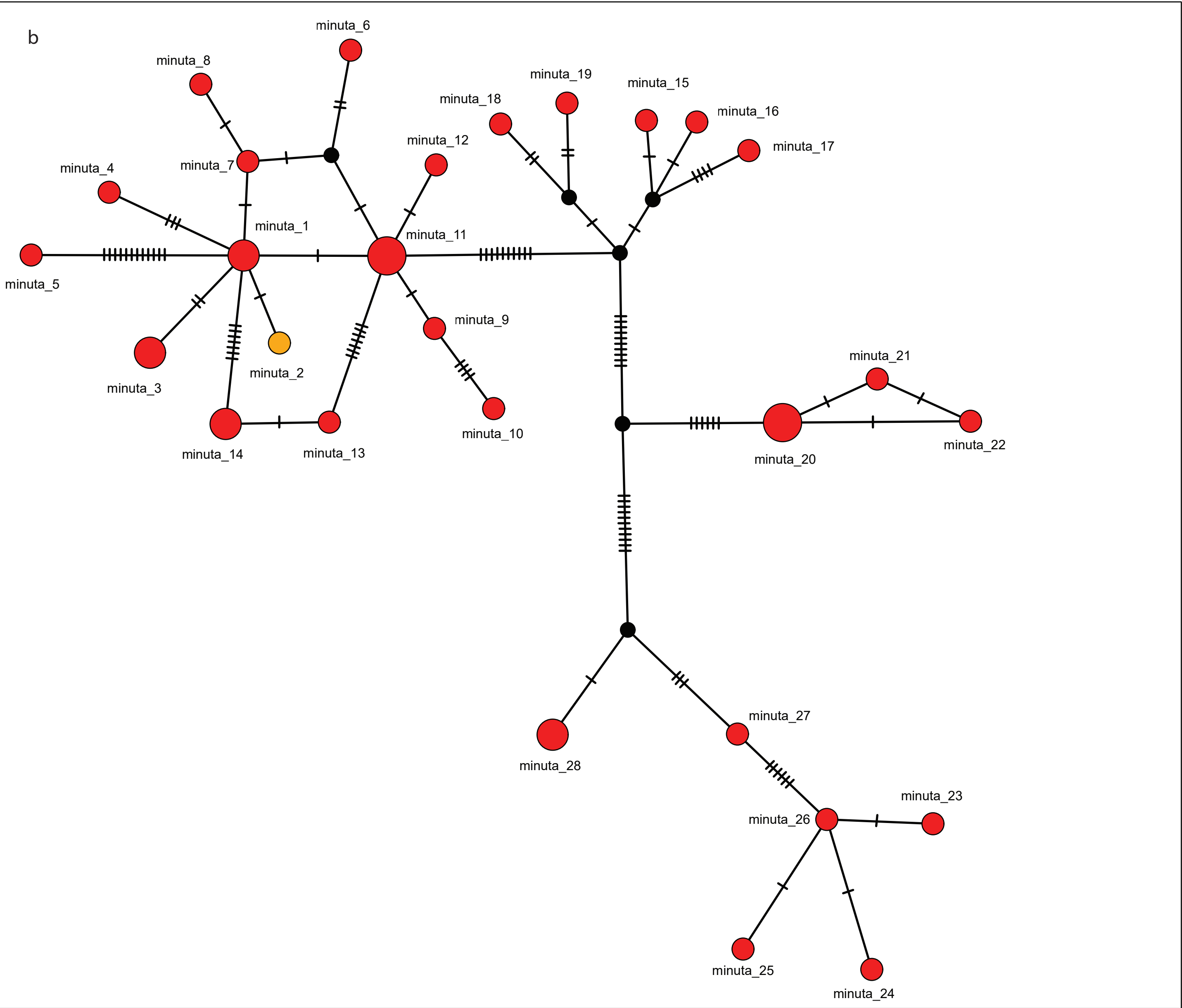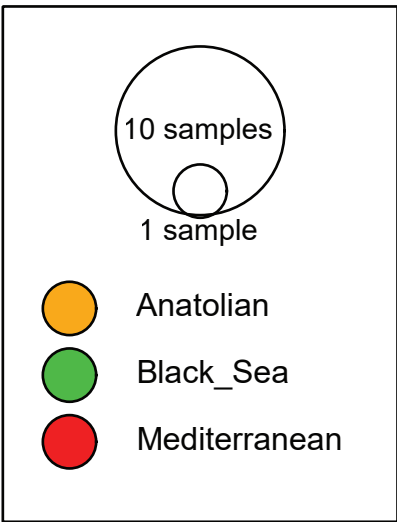

Supplement: Supplementary file 12 — Additional file 12: Figure S10. Haplotype networks obtained for the 62 Sergentomyia specimens analyzed from Turkey (a S. dentata; b S. minuta). Haplotypes are sized according to their relative frequencies and colored by their geographical origin. Missing haplotypes are denoted by small black circles and the numbers of mutational steps are represented by the dashes. [file 13071_2019_3669_MOESM12_ESM.pdf]

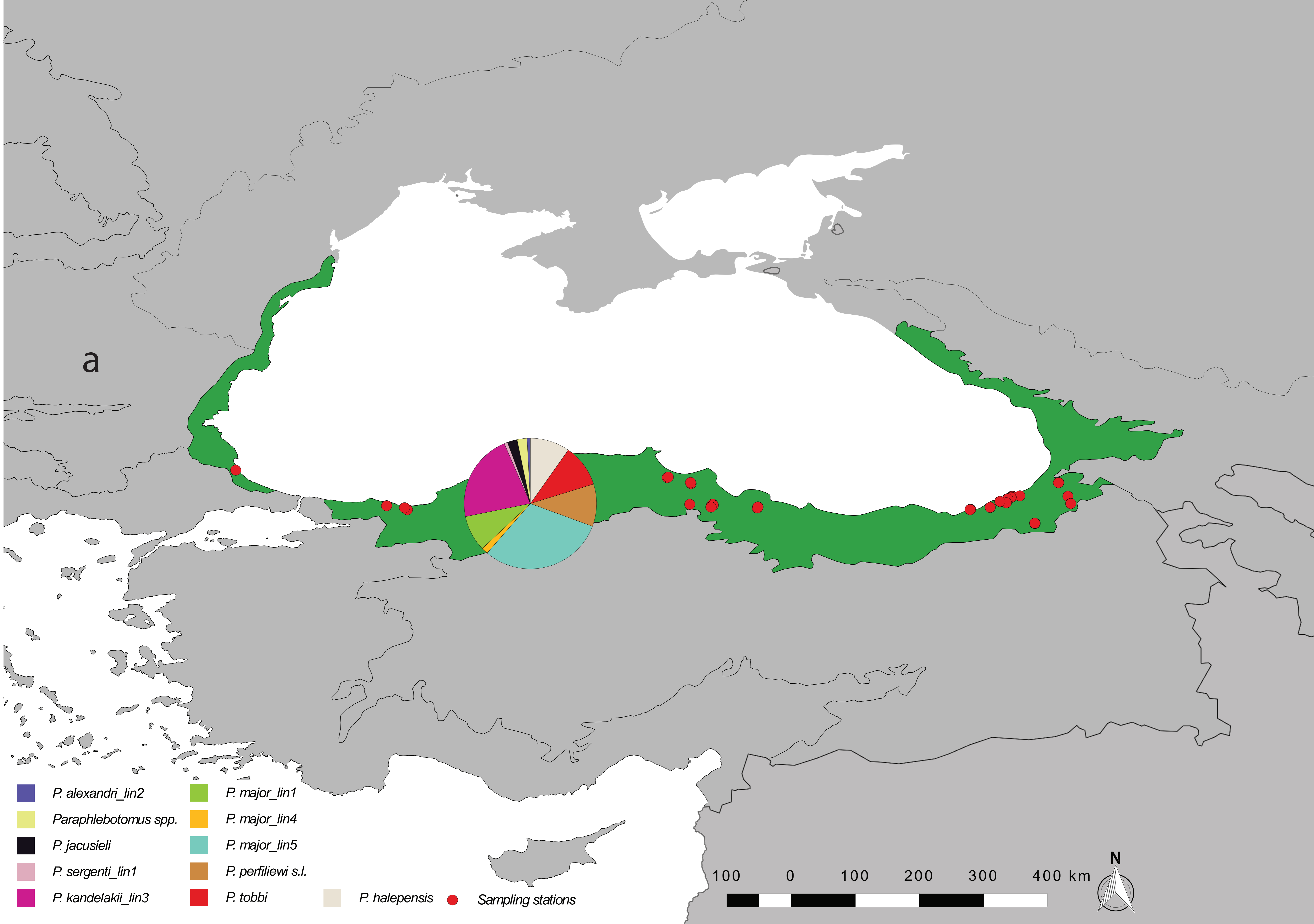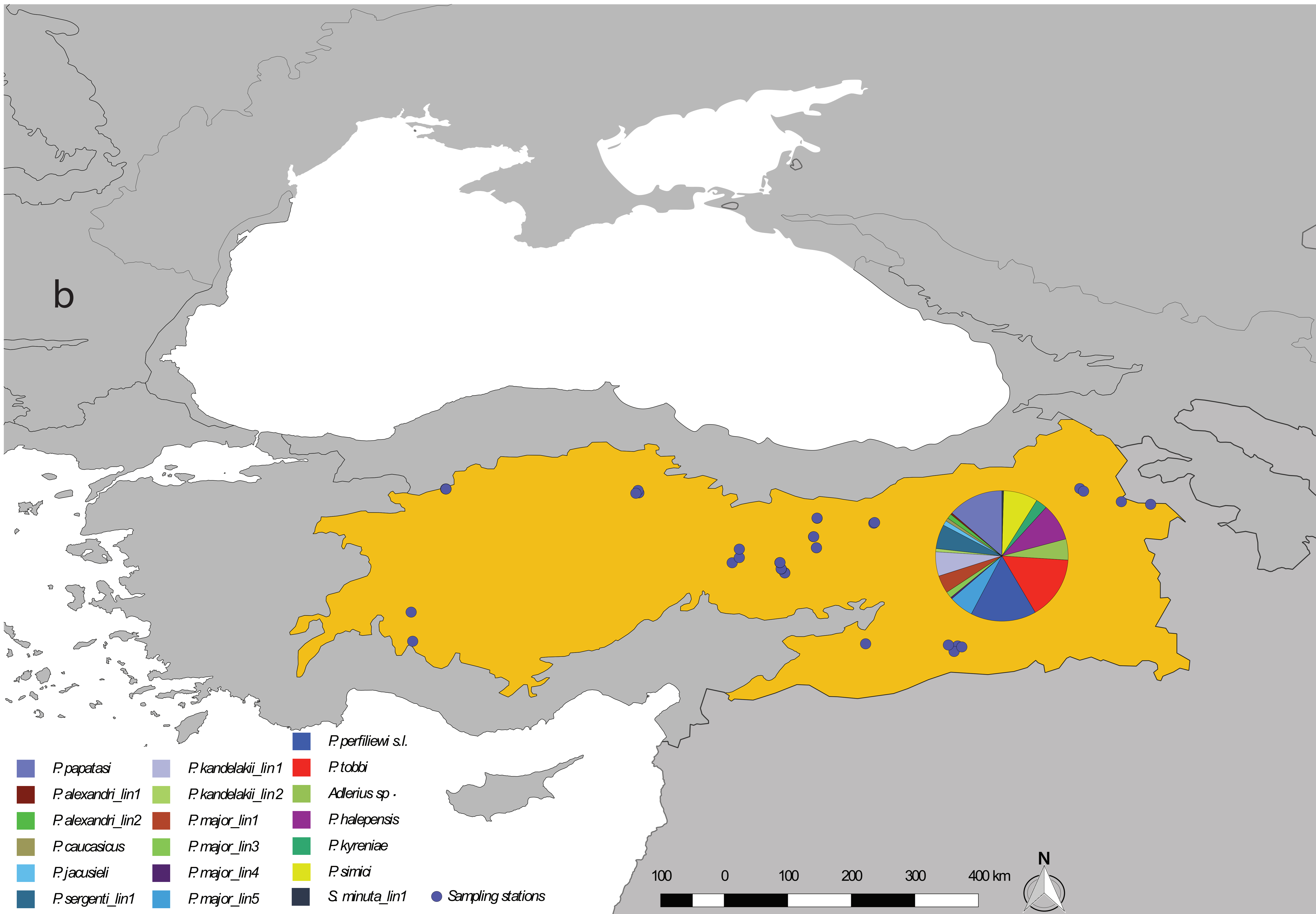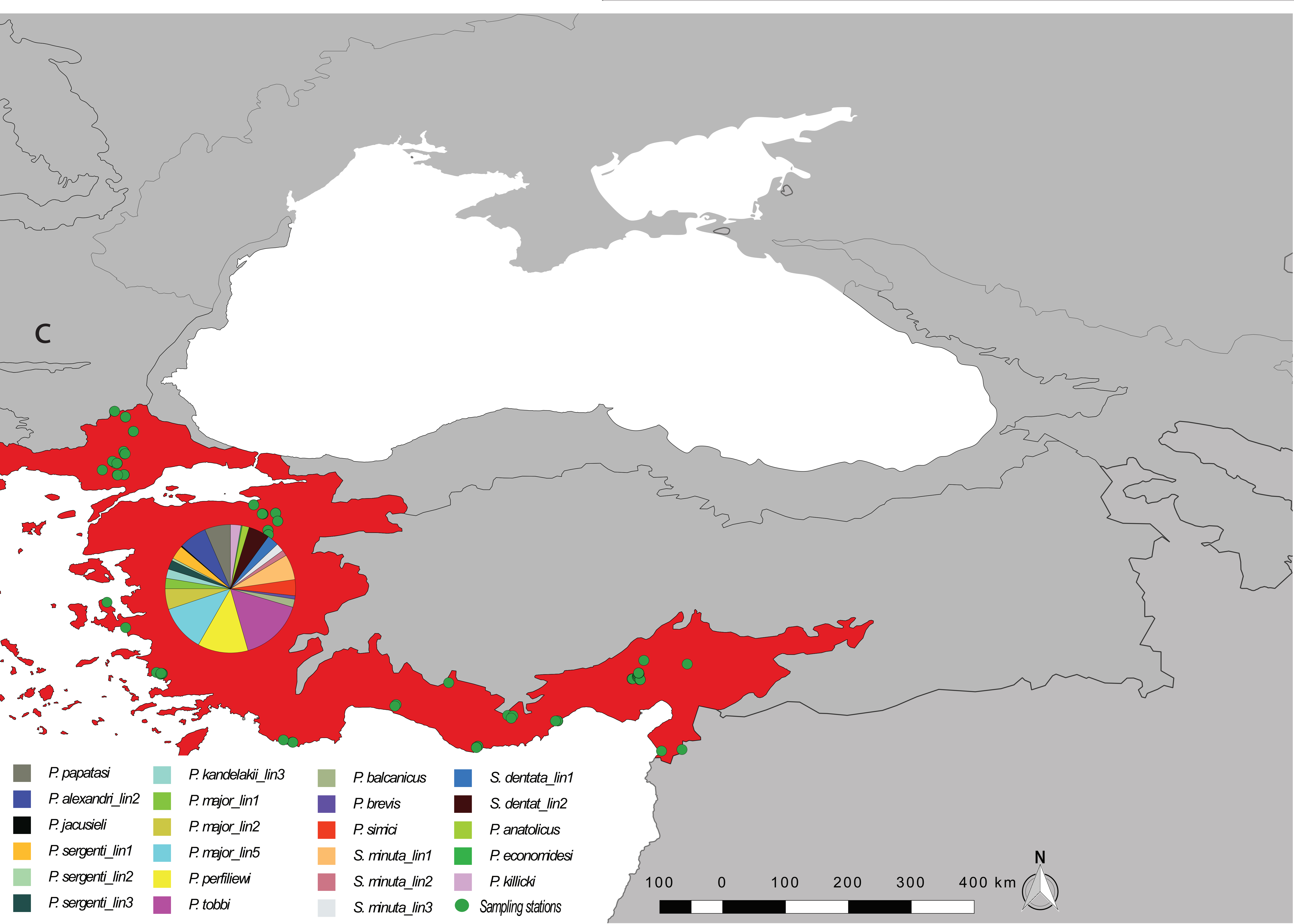

Supplement: Supplementary file 13 — Additional file 13: Figure S11. The distribution of the MOTUs observed in the three biogeographical regions of Turkey. Different colors represent the different sand fly species delineated by all three methods (ABGD, ML and TCS). [file 13071_2019_3669_MOESM13_ESM.pdf]
