# Supplementary material for: Revision of the species composition and distribution of Turkish sand flies using DNA barcodes
Source: Parasit Vectors. 2019 Aug 22;12:410. doi: 10.1186/s13071-019-3669-3 (PMC6704649; doi:10.1186/s13071-019-3669-3)
Supplement: Supplementary file 2 — Additional file 2: Figure S1. Neighbor Joining (NJ) tree using the 741 cox1 sequences obtained for Turkish sand fly specimens. The bootstrap values higher than 50% are indicated. [file 13071_2019_3669_MOESM2_ESM.pdf]

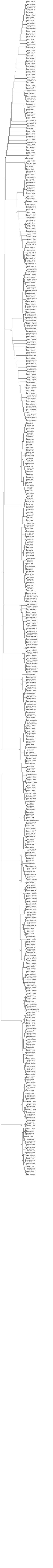
HAM3 P. major s.l.
KOP3 P. major s.l.
KDIS 14 P. major s.l.
KDIS 19 P. major s.l.
HAM6 P. major s.l.
YM2.1 P. major s.l.
HAM5 P. major s.l.
GK2 P. major s.l.
HAM4 P. major s.l.
KOS2.1 P. major s.l.
BLN3 P. major s.l.
UC 2.1 P. major s.l.
BLN2 P. major s.l.
HAM2 P. major s.l.
SFR13 P. major s.l.
NEB5 P. major s.l.
AL1.17 P. major s.l.
AKC 1.32 P. major s.l.
CUM1.5 P. major s.l.
GUN3.1 P. major s.l.
DY3.17 P. major s.l.
YM 1.4 P. major s.l.
DY3.2 P. major s.l.
CAK2 P. major s.l.
BLD7 P. major s.l.
YYL28 P. major s.l.
BLD6 P. major s.l.
CUM3.5 P. major s.l.
BLD5 P. major s.l.
SARI5 P. major s.l.
BLD3 P. major s.l.
KAN2.2 P. major s.l.
IZ1.41 P. major s.l.
BLD2 P. major s.l.
NEB2 P. major s.l.
TEK2.6 P. major s.l.
SARI 3 P. major s.l.
TEK2.4 P. major s.l.
SARI4 P. major s.l.
YAG3.3 P. major s.l.
GUN1.2 P. major s.l.
SFR12 P. major s.l.
TEK1.21 P. major s.l.
YYL27 P. major s.l.
TEK1.3 P. major s.l.
AKC 1.3 P. major s.l.
YES5.2 P. major s.l.
SFR39 P. major s.l.
YES4.2 P. major s.l.
IZ1.37 P. major s.l.
YES3.10 P. major s.l.
IZ2.4 P. major s.l.
YES2.8 P. major s.l.
DEN2.8 P. major s.l.
YES1.15 P. major s.l.
NEB3 P. major s.l.
AKC 1.2 P. major s.l.
NEB4 P. major s.l.
AL3.12 P. major s.l.
YES3.2 P. major s.l.
GAZI2.17 P. major s.l.
KOP2 P. major s.l.
YES1.14 P. major s.l.
BLN4 P. major s.l.
YES1.5 P. major s.l.
IZ3.14 P. major s.l.
GAZI1.1 P. major s.l.
HIZ6 P. major s.l.
HIZ7 P. major s.l.
HIZ10 P. major s.l.
HIZ3 P. major s.l.
KAN2.3 P. major s.l.
HIZ8 P. major s.l.
YES1.2 P. major s.l.
HIZ11 P. major s.l.
YAY21.2 P. major s.l.
DURA3.5 P. major s.l.
YAY18.1 P. major s.l.
BLD4 P. major s.l.
SEY6.8 P. major s.l.
HIZ4 P. major s.l.
SEY4.25 P. major s.l.
YAG2.3 P. major s.l.
SEY2.2 P. major s.l.
GEL1.4 P. major s.l.
SFR38 P. major s.l.
SARI2 P. major s.l.
SEY1.4 P. major s.l.
CAK3 P. major s.l.
ISC21.49 P. major s.l.
YES2.2 P. major s.l.
ISC21.37 P. major s.l.
UC 1.2 P. major s.l.
ISC21.8 P. major s.l.
BLN9 P. major s.l.
ISC20.2 P. major s.l.
DAV2.1 P. major s.l.
KAR1 P. major s.l.
CIC1.2 P. major s.l.
GK4 P. major s.l.
CIC2.10 P. major s.l.
CML3.8 P. major s.l.
KOT78.25 P. major s.l.
CML3.2 P. major s.l.
GK8 P. major s.l.
GK6 P. major s.l.
GK10 P. major s.l.
DUT16 P. major s.l.
CUH12 P. major s.l.
SEB2 P. major s.l.
CUH5 P. major s.l.
TEP3.97 P. major s.l.
OT68 P. major s.l.
SOF95 P. major s.l.
DUT42 P. major s.l.
OT99 P. major s.l.
DUZ23 P. major s.l.
CUH6 P. major s.l.
GED6 P. major s.l.
CUH10 P. major s.l.
CEL12 P. major s.l.
ZER244 P. major s.l.
SEB3 P. major s.l.
SEB5 P. major s.l.
SEB6 P. major s.l.
YUVA1.15 P. major s.l.
KARA14.104 P. major s.l.
KARA 14.108 P. major s.l.
SRG.4 P. major s.l.
TEK1.18 P. major s.l.
KRBRN2 P. major s.l.
GKC2 P. major s.l.
GKC3 P. major s.l.
BAG1.3 P. major s.l.
KOT78.17 P. major s.l.
MAL3.5 P. major s.l.
SEB19 P. major s.l.
SFR14 P. major s.l.
IF15.5 P. major s.l.
IF15.9 P. major s.l.
IF15.10 P. major s.l.
IF15.18 P. major s.l.
IF14.3 P. major s.l.
IF15.17 P. major s.l.
BA61.20 P. major s.l.
YUVA2.5 P. major s.l.
YUVA2.90 P. major s.l.
YUVA1.14 P. major s.l.
YUVA2.14 P. major s.l.
KRBRN4 P. major s.l.
KRBRN44 P. major s.l.
KRBRN45 P. major s.l.
YKOY1.3 P. major s.l.
YYOL5.2 P. major s.l.
ASL5 P. major s.l.
ISK2.2 P. major s.l.
ZFR2.3 P. major s.l.
ISK3.1 P. major s.l.
YYOL7.1 P. major s.l.
BA61.21 P. major s.l.
DERE2 P. major s.l.
IF14.5 P. major s.l.
IF15.2 P. major s.l.
IF15.13 P. major s.l.
IF15.8 P. major s.l.
YUVA1.4 P. major s.l.
YUVA2.15 P. major s.l.
KRBRN3 P. major s.l.
YKOY1.15 P. major s.l.
BOS1.10 P. perfiliewi s.l.
YM1.9 P. perfiliewi s.l.
AL3.17 P. perfiliewi s.l.
COP4.5 P. perfiliewi s.l.
GAZI3.4 P. perfiliewi s.l.
IZ1.2 P. perfiliewi s.l.
BG1.288 P. perfiliewi s.l.
HIS4 P. perfiliewi s.l.
HIS9 P. perfiliewi s.l.
PAM8 P. perfiliewi s.l.
HIS7 P. perfiliewi s.l.
PAM11 P. perfiliewi s.l.
OVA5.1 P. perfiliewi s.l.
BG1.262 P. perfiliewi s.l.
BG2.39 P. perfiliewi s.l.
KDL36 P. perfiliewi s.l.
HCL1 P. perfiliewi s.l.
HCL3 P. perfiliewi s.l.
BLD5.66 P. perfiliewi s.l.
PIR3 P. perfiliewi s.l.
KOT6 P. perfiliewi s.l.
PIR4 P. perfiliewi s.l.
KOT1 P. perfiliewi s.l.
BG1.261 P. perfiliewi s.l.
PAM12 P. perfiliewi s.l.
BLN15 P. perfiliewi s.l.
HIS2 P. perfiliewi s.l.
HIS8 P. perfiliewi s.l.
PAM30 P. perfiliewi s.l.
CAM5.80 P. perfiliewi s.l.
CAM2.69 P. perfiliewi s.l.
CAM2.63 P. perfiliewi s.l.
CAM2.68 P. perfiliewi s.l.
CAM5.46 P. perfiliewi s.l.
KLM2.7 P. perfiliewi s.l.
DAM4.21 P. perfiliewi s.l.
DAM2.69 P. perfiliewi s.l.
CAM2.59 P. perfiliewi s.l.
CAM5.81 P. perfiliewi s.l.
DAM4.29 P. perfiliewi s.l.
BLD5.65 P. perfiliewi s.l.
YAG3.5 P. perfiliewi s.l.
KDL3 P. perfiliewi s.l.
KDL35 P. perfiliewi s.l.
YM1.10 P. perfiliewi s.l.
KDL8 P. perfiliewi s.l.
BG2.34 P. perfiliewi s.l.
YM1.5 P. perfiliewi s.l.
YYL6 P. perfiliewi s.l.
KDL18 P. perfiliewi s.l.
YYL4 P. perfiliewi s.l.
YYL8 P. perfiliewi s.l.
BLN18 P. perfiliewi s.l.
MK2 P. perfiliewi s.l.
HAM3.14 P. perfiliewi s.l.
HAM3.10 P. perfiliewi s.l.
IZ1.4 P. perfiliewi s.l.
GAZI1.3 P. perfiliewi s.l.
IZ1.376 P. perfiliewi s.l.
AL3.8 P. perfiliewi s.l.
KDL31 P. perfiliewi s.l.
YAG3.11 P. perfiliewi s.l.
KDL9 P. perfiliewi s.l.
YAG3.10 P. perfiliewi s.l.
KDL12 P. perfiliewi s.l.
YAG3.6 P. perfiliewi s.l.
BOS1.3 P. perfiliewi s.l.
IZ1.5 P. perfiliewi s.l.
YAG3.4 P. perfiliewi s.l.
BG2.15 P. perfiliewi s.l.
YAG2.7 P. perfiliewi s.l.
MK1 P. perfiliewi s.l.
YAG2.6 P. perfiliewi s.l.
BG2.33 P. perfiliewi s.l.
YAG2.4 P. perfiliewi s.l.
YYL5 P. perfiliewi s.l.
HCL2 P. perfiliewi s.l.
VET3.9 P. perfiliewi s.l.
HCL5 P. perfiliewi s.l.
YAG2.12 P. perfiliewi s.l.
VET3.8 P. perfiliewi s.l.
YM2.4 P. perfiliewi s.l.
VET3.7 P. perfiliewi s.l.
YYL9 P. perfiliewi s.l.
VET3.6 P. perfiliewi s.l.
KDL34 P. perfiliewi s.l.
VET3.5 P. perfiliewi s.l.
YAG2.11 P. perfiliewi s.l.
VET2.1 P. perfiliewi s.l.
VET3.1 P. perfiliewi s.l.
YYL3
YYL10 P. perfiliewi s.l.
IZ1.377 P. perfiliewi s.l.
SARI3.8 P. perfiliewi s.l.
BLD5.29 P. perfiliewi s.l.
IZ1.375 P. perfiliewi s.l.
KRBRN19 P.tobbi
SFR37 P.tobbi
DEN1.10 P.tobbi
AKB5.7 P.tobbi
DEN1.12 P.tobbi
SFR18 P.tobbi
BLD3.18 P.tobbi
SFR3 P.tobbi
DEN1.11 P.tobbi
SFR44 P.tobbi
DEN1.19 P.tobbi
KRBRN49 P.tobbi
KRBRN13 P.tobbi
KRBRN17 P.tobbi
IZ1.3 P.tobbi
YM1.6 P.tobbi
BLD2.2 P.tobbi
BLD5.38 P.tobbi
HAM1.2 P.tobbi
BLD4.14 P.tobbi
AKB2.57 P.tobbi
TURP4.2 P.tobbi
BAK1.43 P.tobbi
IZ1.360 P.tobbi
YM1.11 P.tobbi
AKB5.8 P.tobbi
AKC3.20 P.tobbi
AK3.23 P.tobbi
KOS3.3 P.tobbi
AKC3.24 P.tobbi
KLM1.49 P.tobbi
KDL39 P.tobbi
GK7 P.tobbi
KDL30 P.tobbi
BAS1.2 P.tobbi
KRBRN55 P.tobbi
IM1.64 P.tobbi
BAK1.23 P.tobbi
MK3 P.tobbi
KDL4 P.tobbi
BAK1.31 P.tobbi
KDL6 P.tobbi
AKC2.25 P.tobbi
KDL2 P.tobbi
KDL10 P.tobbi
BAK1.79 P.tobbi
KDL29 P.tobbi
IM3.3 P.tobbi
CUH4 P.tobbi
YUVA1.24 P.tobbi
YLIM3.3 P.tobbi
PAL2.27 P.tobbi
UCP2.4 P.tobbi
TEP4.716 P.tobbi
DRI17 P.tobbi
YUVA1.3 P.tobbi
DRI22 P.tobbi
SEB7 P.tobbi
KKOY1.9 P.tobbi
DRI16 P.tobbi
ASL9 P.tobbi
KLM2.4 P.tobbi
DRI20 P.tobbi
SEB9 P.tobbi
YUVA2.19 P.tobbi
TEK1.22 P.tobbi
TEK1.7 P.tobbi
TEK1.8 P.tobbi
TEK1.4 P.tobbi
TEK1.23 P.tobbi
YYOL4.19 P.tobbi
IZ1.382 P.tobbi
BAK1.80 P.tobbi
KDL7 P.tobbi
DUM1.6 P.tobbi
YLIM2.2 P.tobbi
SEB4 P.tobbi
CAK28 P.tobbi
CUH3 P.tobbi
KDIS2 P.tobbi
CUH5 P.tobbi
CAK27 P.tobbi
BLD5.37 P.tobbi
KKOY1.8 P.tobbi
TEP4.717 P.tobbi
SARI10 P.tobbi
YUVA2.116 P.tobbi
SEB8 P.tobbi
DRI24 P.tobbi
DRI13 P.tobbi
DRI14 P.tobbi
OT5.103 P. P.tobbi
GUN1.5 P.tobbi
DRI21 P.tobbi
OT5.104 P.tobbi
GUN1.4 P.tobbi
YYOL3.46 P.tobbi
DRI15 P.tobbi
BLD4.20 P.tobbi
GAZI2.2 P.tobbi
BLD5.42 P.tobbi
BAG1.24 P.tobbi
YKOY1.27 P.tobbi
GUN1.6 P.tobbi
SARI9 P.tobbi
KLM1.50 P.tobbi
KLM2.3 P.tobbi
BLD100 P. kandelakii s.l.
BLD140 P. kandelakii s.l.
BLD4.10 P. kandelakii s.l.
BLD3.161 P. kandelakii s.l.
BLD11 P. kandelakii s.l.
BLD2.3 P. kandelakii s.l.
BLD125 P. kandelakii s.l.
BLD3.59 P. kandelakii s.l.
BLN14 P. kandelakii s.l.
NEB2.216 P. kandelakii s.l.
YNK2.7 P. kandelakii s.l.
CIC2.1 P. kandelakii s.l.
NEB144 P. kandelakii s.l.
YNK3.8 P. kandelakii s.l.
BLN55 P. kandelakii s.l.
KOS3.2 P. kandelakii s.l.
CIC3.3 P. kandelakii s.l.
DURA3.3 P. kandelakii s.l.
YNK3.9 P. kandelakii s.l.
DURA3.2 P. kandelakii s.l.
YNK2.3 P. kandelakii s.l.
YNK2.8 P. kandelakii s.l.
CIC2.8 P. kandelakii s.l.
NEB80 P. kandelakii s.l.
YNK2.9 P. kandelakii s.l.
NEB31 P. kandelakii s.l.
NEB124 P. kandelakii s.l.
YNK2.11 P. kandelakii s.l.
DURA3.1 P. kandelakii s.l.
NEB2.222 P. kandelakii s.l.
AKC1.21 P. kandelakii s.l.
NEB2.219 P. kandelakii s.l.
NEB114 P. kandelakii s.l.
YNK2.10 P. kandelakii s.l.
YNK3.10 P. kandelakii s.l.
PIRLI2 P. kandelakii s.l.
PIRLI8 P. kandelakii s.l.
BG1.19 P. kandelakii s.l.
KKOY.1.7 P. kandelakii s.l.
KLM1.22 P. kandelakii s.l.
BG1.20 P. kandelakii s.l.
KKOY1.11 P. kandelakii s.l.
BG 1.29 P. kandelakii s.l.
KKOY1.10 P. kandelakii s.l.
KLM1.21 P. kandelakii s.l.
KLM1.24 P. kandelakii s.l.
BG1.28 P. kandelakii s.l.
DUM1.8 P. kandelakii s.l.
KLM.1.28 P. kandelakii s.l.
DUM1.18 P. kandelakii s.l.
YUVA1.16 P. kandelakii s.l.
DUM1.7 P. kandelakii s.l.
DRI27 P. alexandri
KRBRN29 P. alexandri
KDIS2.58 P. alexandri
BLD3.67 P. alexandri
DUM2.20 P. alexandri
AKB2.44 P. alexandri
AKB2.50 P. alexandri
BLD3.89 P. alexandri
AKB2.66 P. alexandri
KRBRN28 P. alexandri
KRBRN63 P. alexandri
SFR69 P. alexandri
DRI11 P. alexandri
SFR32 P. alexandri
BLD3.104 P. alexandri
BG2.208 P. alexandri
TEK2.48 P. alexandri
KDIS2.59 P. alexandri
KDIS2.61 P. alexandri
AKB2.59 P. alexandri
AKB2.43 P. alexandri
BAG1.10 P. alexandri
KDIS2.36 P. alexandri
BLD4.16 P. alexandri
CAK37 P. alexandri
AKB68 P. alexandri
BLD3.127 P. alexandri
BATI7 P. alexandri
BAG1.19 P. papatasi
PAMUKLU3 P. papatasi
DUM1.9 P. papatasi
PAMUKLU4 P. papatasi
KLM2.1 P. papatasi
BG3.7 P. papatasi
BG3.18 P. papatasi
BG3.11 P. papatasi
GAZI1.1 P. papatasi
DUM3.40 P. papatasi
CP3 P. papatasi
CP4 P. papatasi
YUVA1.2 P. papatasi
CP2 P. papatasi
BKVK7 P. papatasi
SRG2 P. papatasi
PNR3 P. papatasi
PAL6 P. papatasi
ZULFIKAR5 P. papatasi
HAC2 P. papatasi
HAC1 P. papatasi
CP1 P. papatasi
ADACIK25 P. papatasi
PAMUKLU2 P. papatasi
DAM4.346 P. papatasi
DAM4.359 P. papatasi
YUVA2.82 P. papatasi
KEL1 P. papatasi
HISIK3 P. papatasi
BKVK3 P. papatasi
KAS2 P. papatasi
BKVK2 P. papatasi
EP2 P. papatasi
PAL1.12 P. papatasi
KRBRN36 P. papatasi
ZULFIKAR4 P. papatasi
KOTEK14 P. papatasi
KRBRN18 P. papatasi
KRBRN32 P. papatasi
YLIM20 P. papatasi
ZULFIKAR12 P. papatasi
TK20-2.3 P. papatasi
DEN1.9 P. papatasi
DEN1.13 P. papatasi
AKB3.6 P. papatasi
AKB3.5 P. papatasi
DEN1.8 P. papatasi
TK32-34.1 P. papatasi
TK32-34.2 P. papatasi
KOTEK8 P. papatasi
KOTEK17 P. papatasi
TK18-2.2 P. papatasi
SFR31 P. papatasi
EP4 P. papatasi
EP1 P. papatasi
EP3 P. papatasi
BLD3.33 P. killicki
BLD5.53 P. killicki
BLD61 P. killicki
BLD63 P. killicki
BLD28 P. killicki
DIK3 P. killicki
BLD.26 P. killicki
KOP17 P. killicki
BLD3.73 Phlebotomus killicki
YES3.17 Adlerius spp.
SFR59 Adlerius spp.
YES2.9 P. simici
YES5.9 Adlerius spp.
YES2.7 Adlerius spp.
YES1.12 Adlerius spp.
YES5.7 P. simici
KDL41 Adlerius spp.
KDL63 Adlerius spp.
KDL75 Adlerius spp.
KDL84 Adlerius spp.
BLD4.23 P. simici
BLD55 P. simici
BLD120 Adlerius spp.
BLD5.21 P. simici
BLD5.31 P. simici
BLD5.34 P. simici
YES1.13 Adlerius spp.
AKB1.4 Adlerius spp.
DEN2.12 P. simici
DEN2.39 Adlerius spp.
DEN2.50 Adlerius spp.
AKB1.3 P. simici
SFR29 P. simici
AYK11 P. simici
BAD2.6 P. simici
CUH7 Adlerius spp.
BAG1.1 P. simici
BAG1.15 Adlerius spp.
BAG1.14 Adlerius spp.
BAG1.6 P. simici
KKOY1.2 P. simici
BAG1.4 P. simici
BAG1.13 Adlerius spp.
CUH13 Adlerius spp.
BAG1.7 P. simici
KKOY1.18 Adlerius spp.
SFR54 Adlerius spp.
BLD4.9 P. brevis
SFR48 Adlerius spp.
BG1.23 Adlerius spp.
BG1.25 Adlerius spp.
DUM1.13 Adlerius spp.
DUM1.16 Adlerius spp.
KLM1.29 Adlerius spp.
KKOY1.16 Adlerius spp.
KKOY1.13 Adlerius spp.
KKOY1.15 Adlerius spp.
KKOY2.22 Adlerius spp.
KKOY3.10 Adlerius spp.
KKOY3.12 Adlerius spp.
KLM1.33 Adlerius spp.
KKOY2.20 Adlerius spp.
BLD4.6 P. anatolicus
BLD5.56 P. anotolicus
BLD3.112 P. anatolicus
BLD3.148 P. anatolicus
BLD4.31 P. anatolicus
BLD5.20 P. anatolicus
BLD5.49 P. economidesi
CUM1.3 P. balcanicus
CUM1.4 P. balcanicus
DAV1.28 Adlerius spp.
CUM1.2 P. balcanicus
HAM2.1 Adlerius spp.
CUM1.10 Adlerius spp.
CUM2.3 Adlerius spp.
DRI7 P. kyreniae
DRI4 P. kyreniae
DRI5 P. kyreniae
DRI3 P. kyreniae
DRI6 P. kyreniae
DRI18 Adlerius spp.
DRI26 Adlerius spp.
BAD2.3 P. halepensis
SRG1 P. halepensis
SRG6 Adlerius spp.
PNR1 P. halepensis
PNR2 P. halepensis
PNR17 Adlerius spp.
DRI41 Adlerius spp.
AK1.2 P. halepensis
ASL10 Adlerius spp.
YKOY1.2 P. halepensis
TEK1.12 Adlerius spp.
CML1.5 P. halepensis
YYOL3.61 Adlerius spp.
KOS1.2 P. halepensis
KOS1.1 P. halepensis
KOS1.3 P. halepensis
CML1.4 P. halepensis
CML1.7 Adlerius spp.
ISK1.2 P. halepensis
KOT3 P. halepensis
KOT2 P. halepensis
KOT7 P. halepensis
KOT13 P. halepensis
TK18.2-1 P. halepensis
TK20-2.1 P. halepensis
TK19-2.1 P. halepensis
KDL5 P. halepensis
BG1.4 P. halepensis
KA2 P. halepensis
YUVA2.4 P. halepensis
KLM1.18 P. halepensis
BG1.21 Adlerius spp.
YUVA1.37 Adlerius spp.
BAD2.28 P. halepensis
DAM4.336 P. jacusieli
GS1 P. jacusieli
YUVA2.76 P. jacusieli
YUVA2.77 P. jacusieli
GS2 P. jacusieli
GS3 P. jacusieli
YUVA2.83 P. jacusieli
TK21-4.2 P. caucasicus
KOT15 P. caucasicus
TEK4.9 Paraphlebotomus spp.
TEK4.4 Paraphlebotomus spp.
TEK4.5 Paraphlebotomus spp.
DY3.15 P. sergenti
DY3.16 P. sergenti
CUM1.19 P. sergenti
DY3.18 P. sergenti
GEL1.2 P. sergenti
GEL2.3 P. sergenti
DAV1.1 P. sergenti
YAG3.2 P. sergenti
DAV1.10 P. sergenti
GEL2.2 P. sergenti
ZER4.1585 P. sergenti
PAL1.56 P. sergenti
HS1 P. sergenti
ZER2.688 P. sergenti
ZER3.368 P. sergenti
KDIS2.66 P. sergenti
YLIM8 P. sergenti
ZER2.628 P. sergenti
YKOY1.26 P. sergenti
HS2 P. sergenti
KOP18 P. sergenti
PIRLI5 P. sergenti
SRG3 P. sergenti
CAK10 P. sergenti
SARI63 P. sergenti
GAZI1.4 P. sergenti
GAZI1.5 P. sergenti
CS1 P. sergenti
CS2 P. sergenti
DUM2.17 P. sergenti
DUM2.18 P. sergenti
GAZI2.1 P. sergenti
DUM2.23 P. sergenti
DUM2.28 P. sergenti
DUM2.21 P. sergenti
DUM2.19 P. sergenti
DUM2.16 P. sergenti
DUM2.22 P. sergenti
BAG1.11 P. sergenti
BAG1.12 P. sergenti
HAM1.5 S. dentata
IZ3.5 S. dentata
AL1.2 S. dentata
SFR68 S. dentata
BOS2.11 S. dentata
IZ2.157 S. dentata
TURK1.14 S. dentata
TURP4.3 S. dentata
IZ2.149 S. dentata
IZ2.168 S. dentata
SFR52 S. dentata
IZ2.173 S. dentata
BLD5.51 S. dentata
BLD4.21 S. dentata
BLD4.40 S. dentata
BLD5.57 S. dentata
KRBRN65 S. dentata
BLD5.52 S. dentata
DAM4.375 S. dentata
ZER2.212 S. dentata
ZER2.211 S. dentata
DAM4.312 S. dentata
ZER2.167 S. dentata
ZER2.210 S. dentata
KDIS2.64 S. dentata
KDIS2.39 S. dentata
KDIS2.42 S. dentata
KDIS2.49 S. dentata
AKB2.76 S. minuta
SFR67 S. minuta
KRBRN52 S. minuta
KRBRN64 S. minuta
SFR66 S. minuta
AKB161 S. minuta
AKB5.9 S. minuta
SFR34 S. minuta
SFR35 S. minuta
SFR62 S. minuta
DIK9 S. minuta
BLD3.163 S. minuta
AKB23 S. minuta
KDIS2.56 S. minuta
BLD4.22 S. minuta
BLD3.168 S. minuta
BLD5.59 S. minuta
BLD4.34 S. minuta
DIK22 S. minuta
BLD3.189 S. minuta
BLD3.156 S. minuta
KDIS2.46 S. minuta
KDIS2.34 S. minuta
KDIS2.44 S. minuta
KDIS14 S. minuta
KDIS2.40 S. minuta
KDIS2.43 S. minuta
BLD3.153 S. minuta
KDIS2.63 S. minuta
KDIS57 S. minuta
BLD5.30 S. minuta
KDIS2.50 S. minuta
PIRLI9 S. minuta
BLD5.50 S. minuta
KDIS2.37 S. minuta
